# A mathematical model for managing the multi-dimensional impacts of the COVID-19 pandemic in supply chain of a high-demand item

**DOI:** 10.1007/s10479-022-04650-2

**Published:** 2022-04-11

**Authors:** Sanjoy Kumar Paul, Priyabrata Chowdhury, Ripon Kumar Chakrabortty, Dmitry Ivanov, Karam Sallam

**Affiliations:** 1 UTS Business School, University of Technology Sydney, Sydney, Australia; 2 School of Accounting, Information Systems and Supply Chain, RMIT University, Melbourne, Australia; 3 School of Engineering and Information Technology, University of New South Wales, Canberra, Australia; 4 Department of Business and Economics, Supply Chain and Operations Management, Berlin School of Economics and Law, Block B, B 3.49, Badensche Str. 50, 10825 Berlin, Germany; 5 School of IT and Systems, The University of Canberra, Canberra, Australia; 6 The Faculty of Computers and Information, Zagazig University, Zagazig, Egypt

**Keywords:** Supply chain resilience, COVID-19 pandemic, Recovery planning, Stochastic modelling, Chance-constrained programming

## Abstract

The COVID-19 pandemic has wreaked havoc across supply chain (SC) operations worldwide. Specifically, decisions on the recovery planning are subject to multi-dimensional uncertainty stemming from singular and correlated disruptions in demand, supply, and production capacities. This is a new and understudied research area. In this study, we examine, SC recovery for high-demand items (e.g., hand sanitizer and face masks). We first developed a stochastic mathematical model to optimise recovery for a three-stage SC exposed to the multi-dimensional impacts of COVID-19 pandemic. This allows to generalize a novel problem setting with simultaneous demand, supply, and capacity uncertainty in a multi-stage SC recovery context. We then developed a chance-constrained programming approach and present in this article a new and enhanced multi-operator differential evolution variant-based solution approach to solve our model. With the optimisation, we sought to understand the impact of different recovery strategies on SC profitability as well as identify optimal recovery plans. Through extensive numerical experiments, we demonstrated capability towards efficiently solving both small- and large-scale SC recovery problems. We tested, evaluated, and analyzed different recovery strategies, scenarios, and problem scales to validate our approach. Ultimately, the study provides a useful tool to optimise reactive adaptation strategies related to how and when SC recovery operations should be deployed during a pandemic. This study contributes to literature through development of a unique problem setting with multi-dimensional uncertainty impacts for SC recovery, as well as an efficient solution approach for solution of both small- and large-scale SC recovery problems. Relevant decision-makers can use the findings of this research to select the most efficient SC recovery plan under pandemic conditions and to determine the timing of its deployment.

## Introduction

The COVID-19 pandemic has severely impacted human lives and disrupted national economies. As of 11 February 2022, more than 407 million people have been infected with COVID-19 and more than 5.8 million people have died (Worldometers, 2022). As a result of the severe impacts of pandemic, the global economy has contracted by 3.5 percent, and according to the Global Economic Prospects report published in June 2021 (The World Bank, 2021), the recession in 2020 was the deepest the world has seen since World War II. This pandemic has also severely impacted businesses and their supply chains (SC). While industry-specific impacts including financial loss, site shutdowns, job cuts, creation of waste and loss of trade relationships have been reported in many articles (Belhadi et al., 2021; Chowdhury et al., 2020), a recent review (Chowdhury, Paul, Kaisar, & Moktadir, 2021) on COVID-19-related studies in the SC discipline summarized 28 disruptive impacts of the pandemic across various SC areas. Moreover, to mitigate the spread of the disease, world governments have been imposing various restrictions on human and economic activities. Consequently, SC operations have been subject to heavy disruptions, where firms have reported interruptions from their SC partners. According to a recent SC resilience report, 84 percent of businesses reported interruptions in their cross border activities and another 70 percent reported disruptions in domestic movements (Elliott, 2021). These disruptions have also occurred beyond tier one SC partners, as the report suggested that 40 percent of COVID-19-related SC disruptions occurred at tier two and beyond. In this unique setting, SC face long-term highly unstable and unpredictable conditions caused by the devastating impacts of the COVID-19 outbreak.

The severe impacts caused by the pandemic pose a unique question around recovery of the SC under extraordinary conditions (Craighead et al., 2020; Golan et al., 2020; Queiroz et al., 2020). Literature has examined SC operations in a pandemic setting as a specific research area (Cheramin et al., 2021; Choi, 2020; Govindan et al., 2020; Ivanov & Dolgui, 2020a; Ivanov, 2020a, 2020b; Kargar et al., 2020; Pamucar et al., 2022; Singh et al., 2020; van Hoek, 2020; Yu et al., 2020). However, the focus of the majority of these studies remains on the analysis of the pandemic impacts (Chowdhury et al., 2021). More specifically, research focusing on developing recovery models and plans is scarce. On the other hand, recovery decisions have been surrounded by multi-dimensional uncertainty stemming from singular and correlated uncertainties in product demand, supply, and production capacities (Dolgui et al., 2020; Aldrighetti et al. 2021; Rozhkov et al. 2022). This emerging area of research is thus related to deep uncertainty and correlated SC processes such as production and logistics (Klibi et al., 2010; Baghalian et al., 2013; Fahimnia et al., 2015; Qazi et al., 2017; Amiri-Aref et al., 2018, 2019; Fathollahi-fard et al., 2019; Snoeck et al., 2019; Zhao & Freeman, 2019; Farahani et al., 2020; Sawik, 2020; Paul, Chowdhury, et al., 2021; Paul et al., 2021).

During the COVID-19 pandemic, many SCs of high-demand items have faced numerous, simultaneous challenges, such as a surge in demand for face masks in global markets, paired with a breakdown of global SCs and a lack of manufacturing capacity (Parker, 2020; Paul et al., 2021; Paul Moktadir, & Ahsan, 2021; Paul, Chowdhury, et al., 2021). Other high-demand products such as sanitizers and face shields have similarly become valuable (and increasingly scarce) commodities during this time (Taylor, 2020). However, due to government-imposed lockdowns, social distancing, and the closing of borders, the supply and manufacturing capacities of such products have been impacted significantly. Moreover, government restrictions have been imposed at different times in different parts of the world. Such timing of restrictions has further impacted SCs, as the timing around the opening and closing of factories of different SC partners represents a key factor determining the impact of global disruptions on SC performance (Ivanov, 2020a, 2020b).

Accordingly, SCs have been facing multi-dimensional disruptions, in which the demand for necessary (i.e., high-demand) items such as face shields and hand sanitizer, the supply of raw materials, and production capacities have become simultaneously vulnerable on an unprecedented scale. Moreover, supply, demand, and production capacities can face different levels of uncertainties and variations, further complicating SC recovery plans. Therefore, developing a recovery plan that considers these multi-dimensional disruptions and uncertainties caused by the COVID-19 pandemic is necessary. In developing the recovery plan, a quantitative modelling approach can account for all these multi-dimensional disruptions and their associated uncertainties. Taking a quantitative approach to develop a recovery plan will not only help to develop a more robust recovery model but will also help SC managers in selecting an optimal recovery plan and associated strategies.

Currently, there are few studies focusing on the optimisation of SC recovery under conditions of severe disruption (Vahdani et al., 2011; Hishamuddin et al., 2013, 2014; Ivanov et al., 2014, 2016a,b; Chen et al., 2015; Shishebori et al., 2017; Azad & Hasini, 2019; Pavlov et al., 2019). Ivanov et al. (2017) conducted a literature review of SC recovery research, which revealed that most studies offer deterministic recovery models with small-scale and single-dimensional disruption in production, supply, or transportation. Accordingly, the research on mathematical recovery models for multi-dimensional disruptions with different levels of uncertainties is still in its infancy. Considering such limitations in the literature, the current study aimed to develop a quantitative recovery model for managing the multi-dimensional uncertainty impacts of the COVID-19 pandemic. Specifically, we proposed the following research objectives:

i. To develop a mathematical model for a three-stage SC to recover from multi-dimensional impacts of the COVID-19 pandemic, such as increased demand, reduced production capacity, and reduced supply capacity;
ii. To consider uncertainties in demand, supply, and production capacities simultaneously; and
iii. To develop an efficient solution approach to solve the model for both small- and large-scale problems.

To achieve these objectives, we developed a chance-constrained programming-based mathematical model with total profit in the recovery window as the objective function. To optimise the recovery plan, we considered several management strategies, such as the emergency supply of raw materials and an increase in production capacity by running extra shifts, hiring more manpower, as well as the cost for demand lost. We assumed that both an emergency supply and an increase in production capacity are also uncertain. Therefore, we developed a unique and enhanced multi-operator differential evolution $(ED{E}_{con}$) variant-based solution approach to solve the model. Through extensive numerical experiments, we demonstrated how the proposed solution approach is capable of efficiently solving both small- and large-scale SC recovery problems.

This study makes several contributions. *First*, we examined a unique problem setting with multi-dimensional uncertainty and demonstrated improvements in efficiency and responsiveness, which can be achieved by mathematical optimisation of SC recovery. Our *second* contribution lies in the conceptualization of a novel model and solution approach that allows for optimal SC recovery. Distinctively, our model is capable of considering different levels of uncertainty in the variations of supply, demand, and production capacities. To the best of our knowledge, our model is the first to consider SC recovery in light of simultaneous demand, supply, and capacity disruptions in a pandemic setting. Our *third* contribution is related to the efficient solution approach of the formulated chance-constrained programming model, which can solve both small- and large-scale SC recovery problems.

The paper is organised as follows. Section 2 discusses the relevant literature streams. The problem description and model formulation are presented in Sect. 3. Section 4 discusses the solution approach. The experiments and discussion of the results are presented in Sect. 5. Finally, the paper concludes with Sect. 6, where a summary of the study’s major findings is given and future research avenues are addressed.

## Literature review

This study contributes to several research streams: the development of an SC recovery model, stochastic modelling of SC disruptions for correlated and large-scale SC disruptions and disruption management in a pandemic setting. Our literature review is organized accordingly and presents the rationale for our study.

### Supply chain recovery modelling

Studies on SC disruptions, referred to as catastrophic events, have received increased attention in the literature (Ivanov et al., 2017). Given these events are difficult to predict and their incidence impossible to eliminate (Christopher et al., 2011), an understanding of how SCs can recover from the negative impacts of these events when they occur is critical. To achieve this, firms must formulate and implement appropriate recovery strategies (Chowdhury et al., 2019).

Mathematical modelling approaches dominate the methodology used in designing such recovery strategies and plans (Baryannis et al., 2019; Duong & Chong, 2020). For example, mathematical recovery models have been developed for supply disruption (Darom et al., 2018; Paul & Rahman, 2018; Paul et al., 2016; Safaeian et al., 2019; Silbermayr & Minner, 2016), production disruption (Ivanov, 2019; Paul et al., 2014a, 2015a, 2015b), demand disruption (Paul et al., 2014b; Rezapour et al., 2011), and scheduling and transportation disruption (Hishamuddin et al., 2013; Paul, Asian, et al., 2019). While a combination of supply and demand disruptions (Ivanov et al., 2014; Sawik, 2019), as well as supply, production, and demand disruptions (Paul, Sarker, et al., 2019) have been considered in modelling the SC recovery, the research on multi-dimensional uncertainties is still scarce. Additionally, existing work on SC disruptions using mathematical modelling mostly ignores the chance-constrained programming approach (CCPA) for dealing with parametric or right-hand side uncertainties.

Although these models and associated strategies and plans are effective for recovering from small-scale disruptions, they are not readily applicable for recovery from a major epidemic or pandemic outbreak. Moreover, pandemic settings are characterised by deep uncertainty worldwide and simultaneous impacts (Ivanov, 2020a). However, the majority of the studies, which appeared in the pre-COVID-19 era, have not considered uncertainty at the level of simultaneous variations in demand, supply, and production when modelling the disruption recovery plan (Paul & Chowdhury, 2021). This study considered such uncertainty, along with the multi-dimensional impacts of the COVID-19 pandemic, which represents a novel and distinct contribution made by this study.

### Stochastic modelling of supply chain disruptions

The modelling of stochastic parameters is well-established in the SC disruption literature (Govindan et al., 2017). As an example, a recent study (Rezapour et al., 2021) used a stochastic modelling approach to develop the best trigger time to implement preparedness and response activities for managing disasters. Different approaches to dealing with stochastic parameters—namely, two-stage stochastic programming (TSSP), robust optimisation (RO) (Pishvaee et al., 2011), and CCPA (Ahmadi & Amin, 2019)-have all been applied to SC disruption problems.

More recently, Tolooie et al. (2020) proposed a two-stage stochastic mixed-integer programming model to ensure a reliable and efficient SC network after considering facility disruptions and demand uncertainties. In their TSSP model, decisions regarding facility allocation under different disruptions were handled in the second stage as a scenario decomposition approach. To deal with the uncertain quality status of returned products, Jeihoonian et al. (2017) instead proposed a TSSP model with a focus on single-period recovery only. With numerical experimentation, they further validated their proposed stochastic model against techniques to handle uncertainties. However, in the case of TSSP, since the variables and constraints are scenario-dependent, the model’s numbers may grow exponentially, leading to solution complexity for larger problems. This sensitivity is arguably a major shortcoming of such an approach (Govindan & Fattahi, 2017).

Meanwhile, RO has been commonly used in the literature, particularly when historical data for uncertainty is scant or parameter distributions are difficult to predict. A detailed discussion of different RO techniques to deal with uncertainties is highlighted in a review by Govindan and Cheng (2018). Meanwhile, Shafiei Kisomi et al. (2016) proposed an RO methodology to deal with uncertain SC configuration and supplier-selection problems. Three different uncertainty settings were used to tackle unknown parameters and were solved using an exact approach embedded with the CPLEX software. Although their approach was effective in dealing with stochastic data, their whole SC model was developed for single-period recovery without concern for inventory management. Moreover, due to the additional complexities of using the RO approach, particularly during the design of the objective function and constraints, their approach could not be employed to solve larger SC models. Prakash et al. (2020) also took the RO approach to deal with supply risks, transportation risks, and uncertain demands. RO is often considered an efficient technique to deal with stochastic data; however, its solutions can be too conservative (Prakash et al., 2020). More specifically, using this technique can lead to either an objective value much worse than the nominal solution or even to the infeasibility of a robust problem (Roos & den Hertog, 2020). This conservatism is prevalent due to the constraint-wise approach of RO and its core assumption that all constraints are challenging for all scenarios in the uncertainty setting (Roos & den Hertog, 2020). This shortcoming applies in particular to SC models when dealing with uncertainties.

The CCPA is a comparatively recent approach that can guide risk-averse decision-makers, even when the stochastic parameters or distribution types are unknown (i.e., if no historical data is available). Unlike the TSSP and RO approaches, CCPA is unique when dealing with parametric uncertainties (Ahmadi & Amin, 2019; see Sect. 3.3 for more details). Moreover, traditional stochastic models often result in a higher number of constraints and decision variables in the mathematical formulation due to the additional complexities arising from the stochastic data. On the contrary, in the design of CCPA, it has one fundamental additional constraint (i.e., the chance constraint) only, which has been proven to be computationally less expensive to solve (Luedtke, 2014). Despite this, the CCPA has not been well-explored with stochastic SC data, particularly data that focuses on disruption recovery (Izadikhah & Saen, 2018). Hence, our work pioneers the application of CCPA to multi-dimensional SC uncertainties.

Some studies on epidemic outbreaks in SC and operations management disciplines have also used stochastic modelling as uncertainties involved in decision-making during a major disruption (Farahani et al., 2020). However, the use of stochastic modelling in the epidemic setting is predominantly limited to investigating disease progression, vaccination prioritisation, quarantine programs and decision support system design for humanitarian SCs (Gupta, Starr, Farahani, & Asgari, 2020). On the other hand, the use of stochastic modelling in developing a recovery model for commercial SCs accounting for a major epidemic or pandemic remains limited.

## Research on COVID-19 in commercial supply chains

Epidemic or pandemic settings are referred to as extraordinary cases of SC disruption management (Ivanov & Dolgui, 2020b; Ivanov, 2020b). However, prior to the COVID-19 pandemic, the literature on SC disruptions concerning these types of incidents had been narrowed to humanitarian SCs (Dasaklis et al., 2012; Queiroz et al., 2020; Farahani et al., 2020; Dubey et al., 2020; Zahedi, Salehi-amiri, Smith, & Hajiaghaei-keshteli, 2021).

A great focus, however, has been to investigate various issues of commercial SCs by considering a pandemic setting since the beginning of COVID-19. Among the various issues investigated, the impacts of the pandemic on various areas of SCs and the development of resilience strategies are the most common themes to come out of published articles (Chowdhury et al., 2021). For example, Ivanov (2020a) discusses both the short- and long-terms impacts of COVID-19 and showed that all SCs could significantly be affected by lockdown and quarantine measures. This pandemic is reported to have had simultaneous impacts on both the demand and supply side of SCs (Chiaramonti & Maniatis, 2020), creating an imbalance between the two (Queiroz et al., 2020; Sharma et al., 2020). Such simultaneous demand and supply-side disruption such as demand surge and shutdown of the operations at suppliers are found to have severe and the highest impact on SC operations and performance during this pandemic (Burgos & Ivanov, 2021). Each of the impacts also has a ripple effect on firm operations and their SC (Ivanov et al., 2019; Li & Zobel, 2020). Moreover, the propagation, i.e., the ripple effect, of disruption is influenced by the combinations of demand, supply and logistics risks (Ghadge et al., 2021). Paul and Chowdhury (2021), meanwhile, discuss how production, supply, and demand are significantly impacted by the pandemic setting. A recent study (Hohenstein, 2022) re-confirms the severe impact of this pandemic on SCs with empirical evidence. To facilitate in analyzing the impacts, Hosseini and Ivanov (2021) develop a method to quantify the impacts of SC disruptions during the COVID-19 pandemic.

While for some products (e.g., garments and luxury items) demand has fallen and their manufacturing capacities remain unused, essential items have been subject to substantial increases in demand, with a shortage of raw materials (Ivanov & Dolgui, 2020a). For example, Paul and Chowdhury (2021) report that in SCs of high-demand items, such as hand sanitizers and medicines, firms are experiencing a surge in demand and critically low supply. Among the various SC players, manufacturers, as well as retailers, are more vulnerable to simultaneous multiple disruptions such as supply, demand, and logistics disruption as a seamless flow of operations is highly critical for manufacturing supply chains (Ghadge et al., 2021). Relationships with suppliers have been affected, as communication is limited to audio and video calls, despite strong collaboration is more important in a crisis (Berndt, 2020; M. Sharma et al., 2022; Spieske et al., 2022). Moreover, the COVID-19 outbreak has caused significant product wastage, including that related to essential, high-demand food products, due to the lack of distribution facilities and the obsolescence of certain capital assets (Dente & Hashimoto, 2020).

Several studies have recommended resilience strategies to minimise the impacts and recover from disruptions caused by the pandemic. These studies also highlight the shortcomings of the current response planning and strategies for ensuring SC resilience (van Hoek, 2020). In the pre-COVID-19 era, specific disruption scenarios were considered in terms of recovery and resilience; however, the uncertainty of major disruptions, including the ‘unknown unknowns,’ was not well-investigated or modelled (Golan et al., 2020). Therefore, recent research has called for new developments in SC recovery to deal with significant outbreaks and ensure resilient and sustainable SCs (Craighead et al., 2020; Jabbour et al., 2020). Accordingly, Ivanov (2020b) calls for a viable SC that is adaptable and structurally changeable enough to react agilely to changes, absorb and recover from the disruption, survive at the time of extraordinary global disruption, and is capable of maintaining consistent, sustainable practices even during a pandemic.

Recent research provides the dimensions and their scales of this important concept, supply chain viability, and demonstrates how this improves SC performance during a disruption (Ruel et al., 2021). This viability concept is further extended to an intertwined SC network, which is a complex and interconnected SC (Ivanov & Dolgui, 2020b). Meanwhile, Ivanov (2021b) develops a framework named AURA (Active Usage of Resilience Assets) that postulates how SC resilience should be considered as an inherent, active, and value-adding component rather than just a mechanism to protect from disruption.

Accordingly, appropriate recovery measures and strategies are required for the viability of SC networks. Ivanov (2021c) provides four adaptation strategies such as intertwining, scalability, substitution, and repurposing to ensure SC viability during a pandemic. In this regard, learning from prior disruption is found as key to reconfiguring SC risk management design (Hohenstein, 2022). Increasing capacity to speed up the production process as well as expanding material supply are critical for the creation of essential drugs and high-demand products during a pandemic situation (Yu, Razon, & Tan, 2020). Therefore, production and SC systems should be sufficiently flexible to accommodate and adjust them to fulfill the increased demand (Ivanov & Das, 2020). Moreover, strong bonding among SC partners is also found as effective to enhance supply chain resilience during a pandemic. For example, Spieske et al., (2022) find that bridging strategies such as buyer–supplier relationships are more effective than buffering strategies. However, complementing both buffering and bridging provides superior resilience during a pandemic like the COVID-19. Similarly, Ivanov (2021a) finds that the conjunction of SC coordination and recovery and gradual ramp-up of production capacity could be effective to exit from this pandemic.

In general, the literature suggests that different resilience strategies are required across various industries and product types as the impacts of the pandemic are different in different industries. Accordingly, researchers investigate resilience strategies by considering particular industries. For example, Paul and Chowdhury (2020) explored strategies for improving the service level of toilet paper supply during COVID-19. Their study suggests that resource sharing among the country’s manufacturers, emergency sourcing, producing basic quality toilet paper, and packing a minimum standard size are effective SC management strategies. Similarly, Belhadi et al. (2021) assessed both short- and long-term resilience strategies for automobile and airline industries; and Chowdhury et al. (2020) explored resilience strategies for the food and beverage industries.

Some of the above studies on the COVID-19 pandemic in SC disciplines have used a mathematical modelling approach. For example, Gupta, Starr, et al. (2020), Gupta, Ivanov, et al. (2020)) used the game theoretic model and investigated how SC disruption timing impacts the pricing decision of substitute products. Similarly, Nagurney (2021) used the same approach to develop a framework to capture the labour constraints in fresh produce products during this pandemic, and Ivanov and Dolgui (2020b) introduced the concept of intertwined supply network (ISN), suggesting that major SC disruptions like the COVID-19 pandemic require resistance at the scale of viability. A mixed-integer linear modelling approach was used to analyze the impact of this pandemic on a drug supply network (Lozano-Diez et al., 2020), to provide an equitable vaccine distribution framework in developing countries (Tavana et al., 2021), and to reconfigure food grain SC networks by considering government guidelines (D. Sharma et al., 2021). A multi-objective mixed-integer linear programming modelling was also used to develop a framework for sustainable, responsive, and resilient mixed SC networks (Vali-Siar & Roghanian, 2022). Another study used a non-constrained linear mathematical modelling approach to develop a production recovery plan for high-demand products (Paul & Chowdhury, 2021). Jha et al. (2021) used an asymptomatic-situation-based model to forecast the effect of epidemic outbreaks on SCs. Karwasra et al., (2021) applied graph theory, along with interpretive structural modeling (ISM), to assess the dairy SC vulnerability during the COVID-19 pandemic, and Lotfi et al. (2022) employed regression-based robust optimization to predict the number of patients during the COVID-19 pandemic.

Some studies also used analytical models using various quantitative approaches. For example, Govindan et al. (2020) proposed a decision support system using a fuzzy inference system for managing the demand for specific healthcare SCs. However, they did not consider the commercial aspects. Choi (2020) similarly built an analytical model to bring services nearer to customers’ homes. Meanwhile, Guan et al. (2020) used the adaptive regional input–output (ARIO) model to investigate the impacts of the pandemic on global SCs, and Rahman et al. (2021) used scenario analysis to investigate the effects of the pandemic on the ship recycling industry.

A recent review article by Chowdhury et al. (2021) reports that only one out of 74 COVID-19 related articles has used a stochastic optimization modelling approach. This study (Mehrotra et al., 2020) used stochastic optimization for managing critical resources during a pandemic. However, more studies using stochastic optimization have appeared recently. For example, Sawik (2022) investigates resilience strategies under ripple effect, and Kenan and Diabat (2022) solve the blood product SC issues during a pandemic using a stochastic optimization approach. However, in general, the use of multi-dimensional disruptions and uncertainties in variations in demand, supply, and production capacities is still limited in this research area. In designing an SC recovery model, our study considered each of these factors. To tackle uncertainties, a CCPA was applied. Our study also developed an efficient solution approach using a multi-operator differential evolution variant-based technique to solve both small- and large-scale problems. Therefore, our study is particularly timely in addressing the research gaps identified above while also guiding practitioners involved in managing high-demand essential items under pandemic conditions.

## Problem description and mathematical formulation

In the following section, we describe the mathematical formulation of the ideal and recovery plans.

### Ideal plan

**Fig. 1:**
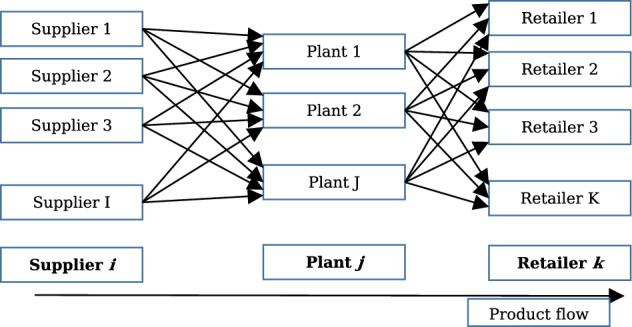
Supply chain network design

In the ideal plan, we considered an SC network system with multiple suppliers, multiple manufacturing plants, and multiple retailers, as presented in Fig. 1.

Such an SC structure is intrinsically linked to high-demand items. For example, in hand sanitizer SCs, manufacturers obtain raw materials (such as isopropyl) from their suppliers. After producing and packaging sanitizers at the manufacturing plants, these are then sent to retailers according to their demands. In this type of SC system, we considered the total production and transportation cost, which was minimised to decide on the transportation, production, and distribution plan (Rezapour et al., 2017; Sawik, 2013).

We introduced the following notations.

$I$Set of suppliers,$\forall i\in I$

$J$Set of manufacturing plants, $\forall j\in J$

$K$Set of retailers,$\forall k\in K$

${S}_{i}$Supply capacity per period of supplier $i$

${P}_{j}$Production capacity per period of manufacturing plant $j$

${d}_{k}$Demand per period of retailer $k$

${T}_{ij}^{1}$Per unit raw material and transportation cost from supplier $i$ to manufacturing plant $j$

${T}_{j}^{2}$Per unit production cost at manufacturing plant $j$

${T}_{jk}^{3}$Per unit transportation cost from manufacturing plant $j$ to retailer $k$

$S$Selling price per unit of product.

In the ideal plan, the decision variables were as follows:

${X}_{ij}$Supply quantity from supplier $i$ to manufacturing plant $j$

${Y}_{j}$Production quantity at manufacturing plant $j$

${Z}_{jk}$Delivery quantity from manufacturing plant $j$ to retailer $k$

The following assumptions were made in the ideal plan:

- There is a single item in the system;
- One unit of the finished product requires one unit of raw material; and
- In the ideal plan, both supply and production capacities are greater than total demand.

In the ideal plan, the objective function is the total profit maximisation, which was calculated as per Eq. (1). The profit function considers revenue, material, transportation, and production cost subject to the constraints presented in Eqs. (2), (3), (4), (5), (6), (7).

Profit per period

1 $$ S\mathop \sum \limits_{j = 1}^{J} \mathop \sum \limits_{k = 1}^{K} Z_{jk} - \left[ {\mathop \sum \limits_{i = 1}^{I} \mathop \sum \limits_{j = 1}^{J} T_{ij}^{1} X_{ij} + \mathop \sum \limits_{j = 1}^{J} T_{j}^{2} Y_{j} + \mathop \sum \limits_{j = 1}^{J} \mathop \sum \limits_{k = 1}^{K} T_{jk}^{3} Z_{jk} } \right] $$

Supply constraint

2 $$ \mathop \sum \limits_{j = 1}^{J} X_{ij} \le S_{i} ; \forall i $$

Balancing between supply and production

3 $$ Y_{j} = \mathop \sum \limits_{i = 1}^{I} X_{ij} ;\forall j $$

Production constraint

4 $$ Y_{j} \le P_{j} ;\forall j $$

Balancing between supply and delivery

5 $$ Y_{j} = \mathop \sum \limits_{k = 1}^{K} Z_{jk} ;\forall j $$

Demand constraint

6 $$ d_{k} = \mathop \sum \limits_{j = 1}^{J} Z_{jk} ;\forall k $$

Non-negativity constraint

7 $$ X_{ij} , Y_{j} , Z_{jk} \ge 0 $$

### Recovery plan

A recovery plan is a revised SC plan for a certain future period (recovery window) based on changed operational parameters (e.g., the impacts of COVID-19) compared with the ideal plan. Our paper outlines the changed parameters used in the current study, as follows:

- Reduced production capacity due to social distancing and other measures, which is uncertain;
- Reduced supply capacity, which is uncertain; and
- Increased demand, which is also uncertain.

We considered a generalised case in which an SC network is affected due to the impact of the COVID-19 pandemic. The demand for some items has increased in an uncertain way, while the shortage of raw material supply has also been uncertain. We further considered demand variation as low, medium, and high (Deblaere et al., 2011). To tackle these uncertainties in supply and demand, the SC plan must be revised to recover from the impacts of COVID-19. The optimal revised SC plan should be generated by adjusting supply, production, and distribution quantities during the recovery window to maximise total SC profit.

To this end, we developed a stochastic mathematical model to generate the recovery plan. To manage the model’s uncertain parameters, we used a chance-constrained method and considered the following recovery strategies in line with the existing literature.

- Increase in production capacity: Using extra shifts, buying machines, utilising spare capacities, hiring manufacturing facilities, and hiring workforce (Paul & Chowdhury, 2020).
- Increase in raw material supply: Using emergency sourcing and collaboration with partners (Dong & Tomlin, 2012; Paul et al., 2021c).
- Cost of demand lost: If an SC is unable to meet demand, there will be a cost for demand losses (Paul et al., 2017).

The following are additional notations for the recovery model.

$N$Number of planning periods in the recovery window.

${p}_{1jn}$Reduced production capacity of plant $j$ at period $n$ in the recovery window, which is uncertain.

${p}_{2jn}$Increase in production capacity of plant $j$ at period $n$ in the recovery window, which is uncertain.

${P}_{jn}^{^{\prime}}$Total production capacity of plant $j$ at period $n$ in the recovery window = ${p}_{1jn}+{p}_{2jn}$.

${S}_{in}^{^{\prime}}$Reduced supply capacity of supplier $i$ at period $n$ in the recovery window, which is uncertain.

$e$Set of emergency suppliers.

${ES}_{en}$Supply capacity of emergency supplier $e$ at period $n$ in the recovery window, which is uncertain.

${\tilde{d }}_{kn}$Increased demand of retailer $k$ at period $n$ in the recovery window, which is uncertain.

${T}_{ijn}^{1}$Per unit raw material and transportation cost from supplier $i$ to manufacturing plant $j$ at period $n$ in the recovery window.

${T}_{jn}^{2}$Per unit production cost at manufacturing plant $j$ at period $n$ in the recovery window.

${T}_{jkn}^{3}$Per unit transportation cost from manufacturing plant $j$ to retailer $k$ at period $n$ in the recovery window.

${T}_{ejn}^{4}$Per unit raw material and transportation cost from emergency supplier $e$ to manufacturing plant $j$ at period $n$ in the recovery window.

${F}_{j}$Fixed cost to increase capacity for manufacturing plant $j$ in the recovery window.

${C}_{j}$Per unit capacity increase cost for manufacturing plant $j$ in the recovery window.

$S$Selling price per unit of product.

In the recovery plan, the decision variables were as follows.

${X}_{ijn}^{^{\prime}}$Supply quantity from current supplier $i$ to manufacturing plant $j$ at period $n$ in the recovery window.

${Y}_{jn}^{^{\prime}}$Production quantity at manufacturing plant $j$ at period $n$ in the recovery window.

${Z}_{jkn}^{^{\prime}}$Delivery quantity from manufacturing plant $j$ to retailer $k$ at period $n$ in the recovery window.

${E}_{ejn}^{^{\prime}}$Supply quantity from emergency supplier $e$ to manufacturing plant $j$ at period $n$ in the recovery window.

Subsequently, we defined the equations for different costs and revenue in the recovery window.

Raw material and transportation cost from current suppliers (RTC_s_)

8 $$ \mathop \sum \limits_{i = 1}^{I} \mathop \sum \limits_{j = 1}^{J} \mathop \sum \limits_{n = 1}^{N} T_{ijn}^{1} X_{ijn}^{^{\prime}} $$

Raw material and transportation cost from emergency suppliers (RTC_e_)

9 $$ \mathop \sum \limits_{e = 1}^{E} \mathop \sum \limits_{j = 1}^{J} \mathop \sum \limits_{n = 1}^{N} T_{ejn}^{4} E_{ejn}^{^{\prime}} $$

Production cost (PC)

10 $$ \mathop \sum \limits_{j = 1}^{J} \mathop \sum \limits_{n = 1}^{N} T_{jn}^{2} Y_{jn}^{^{\prime}} $$

Cost of increasing capacity (CIC)

11 $$ \begin{gathered} \mathop \sum \limits_{j = 1}^{J} F_{j} + \mathop \sum \limits_{j = 1}^{J} C_{j} \left( {\mathop \sum \limits_{n = 1}^{N} Y_{jn}^{^{\prime}} - \mathop \sum \limits_{n = 1}^{N} p_{1jn} } \right) \hfill \\ F_{j} = 0 if\mathop \sum \limits_{n = 1}^{N} Y_{jn}^{^{\prime}} = \mathop \sum \limits_{n = 1}^{N} p_{1jn} \hfill \\ \end{gathered} $$

Transportation cost from manufacturing plants to retailers TC_pr_

12 $$ \mathop \sum \limits_{j = 1}^{J} \mathop \sum \limits_{k = 1}^{K} \mathop \sum \limits_{n = 1}^{N} T_{jkn}^{3} Z^{\prime}_{jkn} $$

Cost of demand lost (CDLL)

13 $$ \left( {\mathop \sum \limits_{k = 1}^{K} \mathop \sum \limits_{n = 1}^{N} \tilde{d}_{kn} - \mathop \sum \limits_{j = 1}^{J} \mathop \sum \limits_{k = 1}^{K} \mathop \sum \limits_{n = 1}^{N} Z^{\prime}_{jkn} } \right) $$

Total revenue (TR)

14 $$ S\mathop \sum \limits_{j = 1}^{J} \mathop \sum \limits_{k = 1}^{K} \mathop \sum \limits_{n = 1}^{N} Z_{jkn}^{^{\prime}} $$

In the recovery plan, the objective function, total profit $\left(TP\right)$ = total revenue – total cost, was determined using Eq. (15).

15 $$ \begin{aligned} Max \left( {TP} \right)\left( {X_{ijn}^{^{\prime}} ,E_{ejn}^{^{\prime}} , Y_{jn}^{^{\prime}} ,Z_{jkn}^{^{\prime}} } \right) & = S\mathop \sum \limits_{j = 1}^{J} \mathop \sum \limits_{k = 1}^{K} \mathop \sum \limits_{n = 1}^{N} Z_{jkn}^{^{\prime}} - \mathop \sum \limits_{i = 1}^{I} \mathop \sum \limits_{j = 1}^{J} \mathop \sum \limits_{n = 1}^{N} T_{ijn}^{1} X_{ijn}^{^{\prime}} - \mathop \sum \limits_{e = 1}^{E} \mathop \sum \limits_{j = 1}^{J} \mathop \sum \limits_{n = 1}^{N} T_{ejn}^{4} E_{ejn}^{^{\prime}} \\ & \quad - \mathop \sum \limits_{j = 1}^{J} \mathop \sum \limits_{n = 1}^{N} T_{jn}^{2} Y_{jn}^{^{\prime}} - \mathop \sum \limits_{j = 1}^{J} F_{j} - \mathop \sum \limits_{j = 1}^{J} C_{j} \left( {\mathop \sum \limits_{n = 1}^{N} Y_{jn}^{^{\prime}} - \mathop \sum \limits_{n = 1}^{N} p_{1jn} } \right) - \mathop \sum \limits_{j = 1}^{J} \mathop \sum \limits_{k = 1}^{K} \mathop \sum \limits_{n = 1}^{N} T_{jkn}^{3} Z_{jkn}^{^{\prime}} \\ & \quad - L\left( {\mathop \sum \limits_{k = 1}^{K} \mathop \sum \limits_{n = 1}^{N} \tilde{d}_{kn} - { }\mathop \sum \limits_{j = 1}^{J} \mathop \sum \limits_{k = 1}^{K} \mathop \sum \limits_{n = 1}^{N} Z_{jkn}^{^{\prime}} } \right) \\ \end{aligned} $$

The objective function (Eq. 15) was subject to the constraints (16)-(23).

Supply constraint from current suppliers

16 $$ \mathop \sum \limits_{j = 1}^{J} X_{ijn}^{^{\prime}} \le S_{in}^{^{\prime}} ; \forall i, n $$

Supply constraint from emergency suppliers

17 $$ \mathop \sum \limits_{j = 1}^{J} E_{ejn}^{^{\prime}} \le ES_{en} ; \forall e, n $$

Production constraints

18 $$ Y_{jn}^{^{\prime}} \le p_{1jn} + p_{2jn} ;\forall j, n $$

Demand constraints

19 $$ \mathop \sum \limits_{j = 1}^{J} Z_{jkn}^{^{\prime}} \le \tilde{d}_{kn} ; \forall k, n $$

Demand loss constraint

20 $$ \mathop \sum \limits_{k = 1}^{K} \mathop \sum \limits_{n = 1}^{N} \tilde{d}_{kn} - { }\mathop \sum \limits_{j = 1}^{J} \mathop \sum \limits_{k = 1}^{K} \mathop \sum \limits_{n = 1}^{N} Z_{jkn}^{^{\prime}} \ge 0 $$

Network balancing constraints

21 $$ Y_{jn}^{^{\prime}} = \mathop \sum \limits_{k = 1}^{K} Z_{jkn}^{^{\prime}} ; \forall j, n $$

22 $$ \mathop \sum \limits_{i = 1}^{I} X_{ijn}^{^{\prime}} = Y_{jn}^{^{\prime}} ; \forall j, n $$

Non-negativity constraints

23 $$ X_{ijn}^{^{\prime}} , Y_{jn}^{^{\prime}} , Z_{jkn}^{^{\prime}} ,E_{ejn}^{^{\prime}} \ge 0; \forall i,j,k,e, n $$

### Chance-constrained programming approach (CCPA)

In this study, we considered a CCPA to deal with uncertain parameters. Based on the study by He et al. (2009), a chance constraint can be symbolised as:

24 $$ Pro\left[ {g\left( {x,\zeta } \right) \ge \beta } \right] \ge \psi $$

where, $\zeta =({\alpha }_{1},{\alpha }_{2},\dots ,{\alpha }_{n})$ is a stochastic vector, the function $g(x,\zeta )$ has the form $g\left(x,\zeta \right)={\alpha }_{1}{x}_{1}+{\alpha }_{2}{x}_{2}+\dots +{\alpha }_{n}{x}_{n}$, and $\beta$ is the maximum value for that function $g\left(x,\zeta \right).$ $\psi$ is the maximum user-given service level (also referred to as a *belief degree*).

Notably, for optimisation problems, uncertainties are broadly categorised into two different forms, namely right-hand-side (RHS) uncertainty and matrix uncertainty (Zhang et al., 2016). Similarly, based on the position of uncertain parameters, constraints in a mathematical model can also be referred to as RHS and left-hand-side (LHS) uncertainties. Essentially, the consideration of uncertainties within a mathematical model or the introduction of stochastic parameters into the optimisation model is acknowledged as a preventive optimisation approach. Consequently, this study also aimed to propose a similarly preventive approach, in which constraints with uncertainties are individually considered with the aid of the CCPA. We assumed that a particular constraint equation of an optimisation problem was $\tilde{A }x\le \tilde{b },$ where $\tilde{A }$ represented matrix uncertainty (i.e., uncertain constraint coefficients $\stackrel{\sim }{{a}_{vc}}$) and $\tilde{b }$ was the RHS uncertainty (i.e., a vector of uncertain parameters). Here, $\forall v\in V$ ($V$ was the number of decision variables for that optimisation problem) and $\forall c\in C$ ($C$ was the number of constraints in that optimization model with uncertainties). Based on Eq. (24), if the model has only RHS uncertainties, the modified chance-constraint equation should take the form of Eq. (25)

25 $$ Pro\left( {\mathop \sum \limits_{v = 1}^{V} a_{cv} x_{v} \le \tilde{b}_{c} } \right) \ge \psi_{c} , c = 1, \ldots ,C $$

To calculate the deterministic equivalent of Eq. (25), it can be reformulated as Eq. (26):

26 $$ \mathop \sum \limits_{v = 1}^{V} a_{cv} x_{v} \le \hat{F}_{{\tilde{b}_{c} }}^{ - 1} \left( {1 - \psi_{c} } \right), c = 1, \ldots ,C $$

where $\hat{F}_{{\tilde{b}_{c} }}^{ - 1} \left( {1 - \psi_{c} } \right)$ is the inverse cumulative density function of RHS uncertain parameters${\tilde{b }}_{c}$, in constraint equation$c$.

### Revised recovery model based on the CCPA

We now explain the process of dealing with uncertain parameters and the revised mathematical model. Due to the impacts of the pandemic, supplier capacity of a supplier $i$ at period $n$ (${S}_{in}^{^{\prime}}$), the production capacity of a manufacturing plant $j$ at period $n$ (${P}_{jn}^{^{\prime}}$), and the supply capacity of an emergency supplier $e$ at period $n$ (${ES}_{en}$) are stochastic in nature.

For the first stochastic parameter,${S}_{in}^{^{\prime}}$, we assumed that $\overline{{S }_{in}^{^{\prime}}}=\left({S}_{1n}^{^{\prime}},\dots ,{S}_{In}^{^{\prime}}\right)\in {\mathfrak{R}}_{n+} \forall n\in N$ is the supply capacity vector for all suppliers $(\forall i\in I)$ at period $n$ in the recovery window. This stochastic vector was assumed to be random with a joint cumulative distribution function (e.g., uniform distribution). Due to this stochastic nature, the supply constraints (16) were no longer well-defined. One possible way to reconfigure these constraints is by considering the most conservative values (minimum) of the supply capacity, which, in contrast, may lead to a product build-up scenario that corresponds to a very unlikely future. The opposite effect is also possible if we consider an optimistic figure for this $\overline{{S }_{in}^{^{\prime}}}$ vector. Thus, considering probabilistic constraints ensures a user-defined threshold. Let us assume ${\theta }_{1}$ is a user-defined upper bound value or credibility level, which ensures that the probability of total supply quantity from a supplier $i$ to manufacturing plant $j (\forall j\in J)$ at period $n$ $(i.e., \sum_{j=1}^{J}{X}_{ijn}^{^{\prime}})$ should be less than or equal ($\le )$ to that of the supplier’s maximum supply capacity, ${S}_{in}^{^{\prime}}$.

Under this assumption, based on the chance-constrained structure as in inequality (26), the deterministic counterpart or equivalent constraint equation of (16) can be re-written, as shown in Eq. (27):

27 $$ \mathop \sum \limits_{j = 1}^{J} X_{ijn}^{^{\prime}} \le \hat{F}_{{S_{in}^{^{\prime}} }}^{ - 1} \left( {1 - \theta_{1} } \right) \forall i \in I, \forall n \in N $$

Thus, to solve this stochastic parameter $\overline{{S }_{in}^{^{\prime}}}$, a decision-maker can use any probabilistic distributions based on previous historical data or any unknown distribution type to fit into Constraint Eq. (26). Based on the confidence level or belief degree ${\theta }_{1}$, a decision-maker will have a different set of constraint equations, which, consequently, will produce a different set of decision variables upon satisfying that confidence level.

Owing to the stochastic nature of capacity for emergency supplier $e$ at period $n$ in the recovery window $({ES}_{en})$, we determined that the stochastic supply capacity vector for all emergency suppliers is $\overline{{ES }_{en}}=\left({ES}_{1n},\dots ,{ES}_{\Gamma n}\right)\in {\mathfrak{R}}_{+}, \forall e\in\Gamma , \forall n\in N$. Here, $\Gamma$ represents the total number of emergency suppliers. Likewise, due to this stochastic nature, Constraint Eq. (17) was ill-defined and, thus, needed to be modified based on the CCPA solely to determine its deterministic counterparts. At this stage, we then assumed that ${\theta }_{2}$ is the user-defined confidence level or belief degree, which ensures that the probability of the total supply quantity from emergency supplier $e$ to manufacturing plant $j$ at period $n$ (${E}_{ejn}^{^{\prime}})$ should be less than or equal to the maximum supplier capacity of emergency supplier $e$ at the period $({ES}_{en})$. Consequently, the deterministic equivalent for Constraint Eq. (17) can be expressed, as shown in Eq. (28):

28 $$ \mathop \sum \limits_{j = 1}^{J} E_{ejn}^{^{\prime}} \le \hat{F}_{{ES_{en} }}^{ - 1} \left( {1 - \theta_{2} } \right) \forall e, n $$

Since the increased production capacity of a manufacturing plant $j$ at period $n$ (${P}_{jn}^{^{\prime}}$) was also considered as a stochastic parameter, we assumed that $\overline{{P }_{jn}^{^{\prime}}}$ is the increased production capacity vector for all plants $j (\mathrm{i}.\mathrm{e}., \forall j\in J)$ at period $n$ in the recovery window, and was defined as$\overline{{P }_{jn}^{^{\prime}}}=\left({P}_{1n}^{^{\prime}},\dots ,{P}_{Jn}^{^{\prime}}\right)\in {\mathfrak{R}}_{+} \forall n\in N$. Due to the stochastic nature of $\overline{{P }_{jn}^{^{\prime}}}$, Constraint Eq. (18) was also not well-defined and needed to be modified based on the abovementioned CCPA. We then assumed that ${\theta }_{3}$ is the user-defined confidence level or belief degree, which ensured that the probability of production quantity at manufacturing plant $j$ at period $n$ in the recovery window $({Y}_{jn}^{^{\prime}})$ was less than or equal to the increased production capacity,${P}_{jn}^{^{\prime}}$. Therefore, the deterministic equivalent for Eq. (18) can be expressed, as shown in Eq. (29):

29 $$ Y_{jn}^{^{\prime}} \le \hat{F}_{{p_{1jn} }}^{ - 1} \left( {1 - \theta_{3} } \right) + \hat{F}_{{p_{2jn} }}^{ - 1} \left( {1 - \theta_{3} } \right) \forall j, n $$

To develop the revised mathematical model for the recovery plan, we replaced all deterministic counterparts based on the CCPA, as explained above. The resultant objective function is presented in Eq. (15) subject to constraints (30)-(37).

30 $$ \mathop \sum \limits_{j = 1}^{J} X_{ijn}^{^{\prime}} \le \hat{F}_{{S_{in}^{^{\prime}} }}^{ - 1} \left( {1 - \theta_{1} } \right) \forall i \in I, \forall n \in N $$

31 $$ \mathop \sum \limits_{j = 1}^{J} E_{ejn}^{^{\prime}} \le \hat{F}_{{ES_{en} }}^{ - 1} \left( {1 - \theta_{2} } \right) \forall e, n $$

32 $$ Y_{jn}^{^{\prime}} \le \hat{F}_{{p_{1jn} }}^{ - 1} \left( {1 - \theta_{3} } \right) + \hat{F}_{{p_{2jn} }}^{ - 1} \left( {1 - \theta_{3} } \right) \quad \forall j, n $$

33 $$ \mathop \sum \limits_{j = 1}^{J} Z_{jkn}^{^{\prime}} \le \tilde{d}_{kn} ; \forall k, n $$

34 $$ \mathop \sum \limits_{k = 1}^{K} \mathop \sum \limits_{n = 1}^{N} \tilde{d}_{kn} - { }\mathop \sum \limits_{j = 1}^{J} \mathop \sum \limits_{k = 1}^{K} \mathop \sum \limits_{n = 1}^{N} Z_{jkn}^{^{\prime}} \ge 0 $$

35 $$ Y_{jn}^{^{\prime}} = \mathop \sum \limits_{k = 1}^{K} Z_{jkn}^{^{\prime}} ; \forall j, n $$

36 $$ \mathop \sum \limits_{i = 1}^{I} X_{ijn}^{^{\prime}} = Y_{jn}^{^{\prime}} ; \forall j, n $$

37 $$ X_{ijn}^{^{\prime}} , Y_{jn}^{^{\prime}} , Z_{jkn}^{^{\prime}} ,E_{ejn}^{^{\prime}} \ge 0; \forall i,j,k,e, n $$

## Solution approach

The differential evolution (DE) algorithm is an evolutionary algorithm utilised to solve optimisation problems through competition and cooperation among solutions within the entire population. DE produces new solutions by following three main operators: mutation, crossover, and selection in each generation. DE-based algorithms are commonly used to solve a wide range of optimisation problems due to their low computational difficulty and strong global search capabilities. Furthermore, they have few control parameters (i.e., population size, scaling factor, and crossover rate) (Tanabe & Fukunaga, 2014). Lastly, DE has been applied in many real-world applications (Wang et al., 2012b; Li et al., 2018; Wang et al., 2012a; Lieckens & Vandaele, 2016).

Although several DE-based algorithms have been developed to obtain the optimal or near-optimal solution for optimisation problems, no single algorithm or search operator has consistently performed as the best solution (Elsayed et al., 2011; Mallipeddi et al., 2011). Therefore, several authors have developed innovations that use more than one evolutionary algorithm (EA) or search operator in a single algorithmic system—referred to as multi-methods or multi-operators—to tackle this issue (Wu et al., 2018; Ali et al., 2015; Sallam et al., 2017). Nevertheless, an effective algorithm for a better, faster, and more reliable solution to the complex problem can still be developed. We thus introduced an enhanced multi-operator DE variant, called *EDE*_*con*_, that uses several DE mutation operators in a single algorithmic system to solve the problem under study.

### EDE__con_ algorithm

The proposed *EDE*_*con*_ algorithm began with the generation of initial random population size (PS) solutions (i.e., X, Y, Z, and E were randomly generated) to ensure that every decision variable lay within its bounds. Then, the objective function value (TP) and total constraint violation were computed for each solution. The number of function evaluations was simultaneously updated. As the proposed algorithm uses several operators (${n}_{op}$), initially, each DE operator was used to guide the same number of solutions $N{S}_{op}$ toward the optimal one. The objective function value and constraint violations for the newly generated populations were computed, followed by pairwise comparisons between each individual in the parent population and its counterpart in the offspring population (discussed in subsection 4.5), with the better of the two joining the next population. At the end of each generation, the improvement value was subsequently computed (discussed in subsection 4.3), based on which $N{S}_{op}$ was updated. A minimum number of individuals updated by each DE mutation strategy was also set (i.e., the population number, which should be updated at each evolutionary stage during the DE mutation, was defined before the algorithm was allowed to run). Indeed, a population size reduction mechanism was used at the end of each iteration by removing the worst individual (Tanabe & Fukunaga, 2014), as in Eq. (45). This was done to preserve diversity at the early generations and boost the convergence in the later ones. The main steps of the proposed $ED{E}_{con}$ algorithm are presented in Algorithm 1.


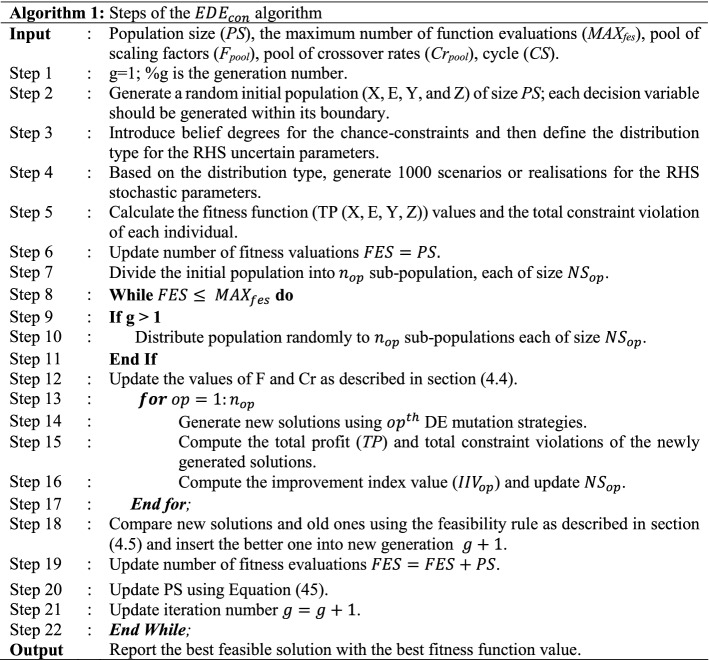


The details of the proposed algorithm are presented in the following subsections.

### DE mutation operators

To guide the whole population to the optimal or near-optimal solutions, we used the following two DE mutation strategies:

- DE/current-to-$\phi$ best/1/bin with archive
  38 $$u_{{i,j}}  = \left\{ {\begin{array}{ll}    {x_{{i,j}}  + F_{i} \left( {x_{{\phi ,j}}  - x_{{i,j}}  + x_{{r1,j}}  - x_{{r2,j}} } \right)} & {if\left( {rand \le Cr_{i} orj = j_{{rand}} } \right)}  \\    {x_{{i,j}} } & {otherwise}  \\   \end{array} } \right.$$
- DE/current-to-$\phi$ best/1/bin without archive
  39 $$ u_{{i,j}}  = \left\{ {\begin{array}{ll}    {x_{{i,j}}  + F_{i} \left( {x_{{\phi ,j}}  - x_{{i,j}}  + x_{{r1,j}}  - x_{{r3,j}} } \right)} & {if\left( {rand \le Cr_{i} orj = j_{{rand}} } \right)}  \\    {x_{{i,j}} } & {otherwise}  \\   \end{array} } \right. $$

where r1, r2, r3 are three mutually exclusive random numbers; ${\overrightarrow{x}}_{r1}$ and ${\overrightarrow{x}}_{r3}$ were randomly chosen from the entire population; and ${\overrightarrow{x}}_{\phi }$ was selected randomly from the best 25% of solutions in the entire population. ${\overrightarrow{x}}_{r2}$ was randomly picked from the union of the whole population and archive, which we used in this study to preserve the population’s diversity. New candidates that are worse than their parent ones were kept in the archive (Zhang & Sanderson, 2009). To make space for the newly generated individuals, once the archive size was greater than its default size, the worst individuals were removed from it.

### Updating ${\varvec{N}}{{\varvec{S}}}_{{\varvec{o}}{\varvec{p}}}$

We used the population diversity and quality of the solutions to determine the number of solutions each DE operator evolved. Considering the population diversity, at the end of each generation ($$g$$), the diversity obtained from each DE search operator ($${D}_{op})$$ was calculated by

40 $$ D_{op} = \frac{{\mathop \sum \nolimits_{i}^{{NS_{op} }} \left( {dis\left( {\overrightarrow {Sol}_{op,i} - \overrightarrow {Sol}_{op}^{best} } \right)} \right)}}{{NS_{op} }}, \forall op = 1,2, \ldots , n_{op} $$

where $dis\left({\overrightarrow{Sol}}_{op,i}-{\overrightarrow{Sol}}_{op}^{best}\right)$ is the Euclidean distance between the ${i}^{th}$ solution and the best one generated by each DE operator. Then the diversity ratio was calculated by

41 $$ DR_{op} = \frac{{D_{op} }}{{\mathop \sum \nolimits_{op = 1}^{{n_{op} }} D_{op} }}, \forall op = 1,2, \ldots ,n_{op} $$

Similarly, for the quality of solutions, the best obtained objective function value (total profit) from each DE operator at generation $g$ was used to calculate the normalised quality of solution (${NQ}_{op}$), as

42 $$ NQ_{op} = \frac{{TP_{g,op}^{best} }}{{\mathop \sum \nolimits_{op = 1}^{{n_{op} }} TP_{g,op}^{best} }}, \forall op = 1,2, \ldots ,n_{op} $$

where $T{P}_{g,op}^{best}$ was the best total profit value obtained by DE operator $op$ at generation $g$. As we were solving a constrained optimisation problem, $T{P}_{g,op}^{best}$ was determined based on both the fitness function values and the total constraint violations. This was done by sorting the solutions based on both fitness function values and total constraint violations.

Based on both the quality of solutions and diversity of the population mentioned above, the improvement index value (IIV) was computed by

43 $$ IIV_{op} = DR_{op} + \left( {1 - NQ_{op} } \right), \forall op = 1,2, \ldots ,n_{op} $$

Finally, the number of solutions that each DE operator will evolve was calculated as

44 $$ NS_{op} = max\left( {0.1,min\left( {0.9,\frac{{IIV_{op} }}{{\mathop \sum \nolimits_{op = 1}^{{n_{op} }} IIV_{op} }}} \right)} \right) \times PS, \forall op = 1,2, \ldots ,n_{op} $$

As one of the DE operators may behave differently during the stages of the optimisation process (i.e., it may perform well at the early stages and worse at the later ones), we used a minimum value (0.1) to provide opportunity for the low-performing operators to be applied.

### Updating the values of *PS*,* F,* and *Cr*

As outlined in the literature, the performance of the DE algorithm is highly dependent on the values of the control parameters. However, setting their values is not an easy task. Researchers have traditionally used a trial-and-error method to determine the best values for the control parameters, which is both tedious and time-intensive. To overcome this challenge, we used a linear population size reduction mechanism to gradually reduce the population size. It begins with a large number of solutions, and over the generations, it gradually decreases until it reaches a minimum number. This is done to maintain population diversity at the early stages of the optimisation process and to boost the convergence at the later stages. The following equation was used to achieve this:

45 $$ PS_{g + 1} = round\left[ {PS^{init} + \left( {\frac{{PS^{min} - PS^{init} }}{{MAX_{FES} }}} \right) \times FES} \right] $$

where $P{S}^{init}$ is the initial population size, $P{S}^{min}$ the minimum population size, *FES* is the current number of fitness evaluations, and $MA{X}_{FES}$ is the maximum number of *FES*.

To update the values of F and Cr, the same mechanism was used (Elsayed et al., 2016; Sallam et al., 2019):

- A historical memory that has *n* components for recording both $MF$ and $MCr$ was initialised with 0.5 for all elements.
- Each solution ${\overrightarrow{Sol}}_{i}$ was linked with its own ${F}_{i}$ and ${Cr}_{i}$ values, such that
  46 $$ F_{i} = randc_{i} \left( {MF_{ri} ,0.1} \right) $$
  47 $$ Cr_{i} = randn_{i} \left( {MCr_{ri} ,0.1} \right) $$
  where ${r}_{i}$ was randomly chosen from $[1, n]$, $rand{c}_{i}$ and $rand{n}_{i}$ are values that were randomly generated by following Cauchy and Normal distributions with variance 0.1, and mean $MF$ and $MCr$, respectively.
- At the end of each generation $g$, the values of ${F}_{i}$ and $C{r}_{i}$ utilized by the successful solutions (i.e., the individuals whose new TP was better than their older ones) were saved in *SF* and *SCr*. The entries of the historical memories were updated as
  48 $$ MF_{t} = mean_{WL} \left( {SF} \right) if SF \ne \phi $$
  49 $$ MCr_{t} = mean_{WA} \left( {SCr} \right) if SCr \ne \phi $$

where $1\le t\le n$ is the position of the element that should be updated in the memory. The initial value of *t* was set to 1 and then increased when a new element was recorded in the memory. If the size of the memory was larger than *n*, it was reset to 1. The Lehmer mean ($mea{n}_{WL}\left(SF\right)$) and weighted mean ($mea{n}_{WA}\left(SCr\right)$) were computed as

50 $$ mean_{WL} \left( {SF} \right) = \frac{{\mathop \sum \nolimits_{\vartheta = 1}^{{\left| {SF} \right|}} \omega_{\vartheta } \times SF_{\vartheta }^{2} }}{{\mathop \sum \nolimits_{\vartheta = 1}^{{\left| {SF} \right|}} \omega_{\vartheta } \times SF\_\vartheta }} $$

51 $$ mean_{WA} \left( {SCr} \right) = \mathop \sum \limits_{\vartheta = 1}^{{\left| {SCr} \right|}} \omega_{\vartheta } \times SCr_{\vartheta } $$

where $\left|SF\right|$ and $\left|SCr\right|$ were the number of successful *F* and *Cr* recorded in *SF* and *SCr* with $\left|SF\right|=|SCr|$, respectively, and ${\omega }_{\vartheta }$ was the weight computed by

52 $$ \omega_{\vartheta } = \frac{{\tau_{\vartheta } }}{{\mathop \sum \nolimits_{\vartheta = 1}^{{\left| {SCr} \right|}} \tau_{\vartheta } }} $$

As the problem of interest is a constrained optimisation problem, the values of ${\tau }_{\vartheta }$ were computed using the following three scenarios.

- Feasible solution to feasible solution: $\varphi \left({\overrightarrow{Sol}}^{best}\right)=0$ at both generations $g-1$ and, where $\varphi \left({\overrightarrow{Sol}}^{best}\right)$ is the violation of the best solution.
- Infeasible to feasible: ${\overrightarrow{Sol}}^{best}$ is infeasible at generation $g-1$ and then becomes feasible at generation $g$.
- Infeasible to infeasible: ${\overrightarrow{Sol}}^{best}$ is infeasible in both generations $g-1$ and $g$.

First, for every successful individual ($t\in \mathrm{1,2},\dots , |SF|$) that fell under scenario (a), its ${\tau }_{\vartheta }$ was computed as

53 $$ \tau_{t} = {\uptheta }_{{\text{t}}} = {\text{max}}\left( {0,\frac{{\varphi_{t,g - 1} - \varphi_{t,g} }}{{\varphi_{t,g - 1} }}} \right) + max\left( {0,\frac{{TP_{t,g - 1} + TP_{t,g} }}{{TP_{t,g - 1} }}} \right) $$

Then, for each successful candidate solution that existed in either scenario (b) or (c), its ${\tau }_{\vartheta }$ was calculated by

54 $$ \tau_{t} = \max \left( {0,{\uptheta }_{{\text{t}}} } \right) + \frac{{\varphi_{t,g - 1} - \varphi_{t,g} }}{{\varphi_{t,g - 1} }} + max\left( {0,\frac{{TP_{t,g - 1} + TP_{t,g} }}{{TP_{t,g - 1} }}} \right) $$

### Constraints handling method

To deal with constrained optimisation problems using an evolutionary algorithm, a constrained handling technique is required to compare the solutions between the parent population and the offspring one. In the current study, the technique proposed by Deb (2000) was used, which has three cases. Suppose we have two solutions, ${\overrightarrow{Sol}}_{old}$ and ${\overrightarrow{Sol}}_{new}$.

- If both ${\overrightarrow{Sol}}_{old}$ and ${\overrightarrow{Sol}}_{new}$ are feasible and$TP\left({\overrightarrow{Sol}}_{new}\right)<TP({\overrightarrow{Sol}}_{old} )$, then ${\overrightarrow{Sol}}_{new}$ is chosen.
- If both ${\overrightarrow{Sol}}_{old}$ and ${\overrightarrow{Sol}}_{new}$ are infeasible and $\varphi \left({\overrightarrow{Sol}}_{new}\right)<\varphi \left({\overrightarrow{Sol}}_{old}\right)$, then ${\overrightarrow{Sol}}_{new}$ is chosen.
- If ${\overrightarrow{Sol}}_{new}$ is feasible and ${\overrightarrow{Sol}}_{old}$ is infeasible, then ${\overrightarrow{Sol}}_{new}$ is preferred.

The total constrained violation for a solution was computed using the following:

55 $$ \varphi \left( {\overrightarrow {Sol} } \right) = \mathop \sum \limits_{k = 1}^{K} {\text{max}}(0,g_{k} \left( {\overrightarrow {Sol} } \right) + \mathop \sum \limits_{e = 1}^{E} {\text{max}}\left( {0,\left| {h_{e} \left( {\overrightarrow {Sol} } \right)} \right| - \Delta_{e} } \right) $$

where ${g}_{k}(\overrightarrow{Sol})$ and ${h}_{e}(\overrightarrow{Sol})$ are the *kth* inequality and *eth* equality constraints, respectively. For each equality constraint ${h}_{e}$, ${\Delta }_{e}$ was set to a value of 0.0001.

## Computational experiments and discussion of results

To validate the proposed $ED{E}_{con}$ algorithm and to demonstrate the efficacy of the proposed SC recovery model, this section discusses the computational experiments for both the ideal and recovery models. The proposed $ED{E}_{con}$ algorithm was coded and implemented into MATLAB R2018b and ran on a PC with 16 GB RAM, core i7 processor with a 3.4 GHz and Windows10. The algorithm was executed 30 times and the average result was recorded based on these 30 runs. As the data was randomly generated, each run can be considered as an instance. To substantiate the proposed model, four different problem sets were considered with varied problem parameters (i.e., number of suppliers, plants, and retailers). Without losing sight of practical reality, we created a generalised dataset, which is common for many real SC designs in high-demand item production. While these are certainly different in detail, they do share a common set of attributes, such as a multi-stage structure and simultaneous disruptions in supply, demand, and capacity. For instance, a problem set 15 × 7 × 15 × 8 × 2 indicates that there are 15 different suppliers (I), 7 different manufacturing plants (J), 15 retailers (K), 8 emergency suppliers (e)$,$ and 2 planning periods (N) in the recovery window. Notably, for the ideal model, similar problem sets were developed, excluding the number of emergency suppliers and planning periods. Table 1 highlights all key input parameters that were hypothetically chosen to solve the proposed models using our EDE__con_ meta-heuristic approach.

**Table 1:** Hypothetical values of some parameters defined to solve both the ideal and recovery models

Input data (parameters)	Problem sets			
	3×2×5×2×4	8×4×10×4×2	10×6×15×6×3	15×7×15×8×2
Supplier ($I$)	3	8	10	15
Plant ($J$)	2	4	6	7
Retailer ($K$)	5	10	15	15
Selling price ($$S$$)	$40/unit	$40/unit	$40/unit	$40/unit
*Ideal Model*				
Supply capacity for each supplier $i$ (${S}_{i}$)	1800 pcs	1800 pcs	1800 pcs	1800 pcs
Production capacity for each plant $j$ (${P}_{j}$)	3000 pcs	3000 pcs	3000 pcs	3000 pcs
Demand for each retailer $k$ (${d}_{k}$)	1000 pcs	1000 pcs	1000 pcs	1000 pcs
$${T}_{ij}^{1}$$	$5/unit	$5/unit	$5/unit	$5/unit
$${T}_{j}^{2}$$	$10/unit	$10/unit	$10/unit	$10/unit
$${T}_{jk}^{3}$$	$2/unit	$2/unit	$2/unit	$2/unit
*Recovery Model*				
Number of planning periods ($N$)	4	2	3	2
Reduced production capacity for each plant and for each period ${(p}_{1jn})$	~ Uniform (0, 3000)	~ Uniform (0, 3000)	~ Uniform (0, 3000)	~ Uniform (0, 3000)
Increase in production capacity for each plant and for each period (${p}_{2jn}$)	~ Uniform (400, 600)	~ Uniform (400, 600)	~ Uniform (400, 600)	~ Uniform (400, 600)
Reduced supply capacity for each plant and for each period (${S}_{in}^{^{\prime}}$)	~ Uniform (0, 1800)	~ Uniform (0, 1800)	~ Uniform (0, 1800)	~ Uniform (0, 1800)
Emergency supplier ($E$)	2	4	6	8
Supply capacity for each $$E$$ ($${ES}_{en})$$	~ Uniform (0, 1500)	~ Uniform (0, 1500)	~ Uniform (0, 1500)	~ Uniform (0, 1500)
*Stochastic demand variability*	(Low, medium, high)			
$${T}_{ijn}^{1}$$	$5/unit	$5/unit	$5/unit	$5/unit
$${T}_{jn}^{2}$$	$10/unit	$10/unit	$10/unit	$10/unit
$${T}_{jkn}^{3}$$	$2/unit	$2/unit	$2/unit	$2/unit
$${T}_{ejn}^{4}$$	$8/unit	$8/unit	$8/unit	$8/unit
$${F}_{j}$$	$1000	$1000	$1000	$1000
$${C}_{j}$$	$5/unit	$5/unit	$5/unit	$5/unit
Lost sales cost $$(L)$$	$20/unit	$20/unit	$20/unit	$20/unit
${\theta }_{1}$	(0.95, 0.90, 0.85)			
${\theta }_{2}$	(0.95, 0.90, 0.85)			
${\theta }_{3}$	(0.95, 0.90, 0.85)			

Given the essential assumptions of this model, production capacities and supply capacities for both regular and emergency suppliers are uncertain due to the global pandemic. As can be observed from Table 1, we arbitrarily used uniform distribution to generate random numbers for handling these uncertain values. One may argue with the distribution selection, as indisputably, the uniform distribution may not mirror the real-life scenario. Indisputably, the prime focus of this study was to demonstrate how a typical SC recovery model works under uncertain environments, and thus, the selection of uniform distribution was solely to exemplify the model parameters. Moreover, to deal with the RHS uncertain parameters in the mathematical optimisation approach, three sets of belief degrees were employed for ${\theta }_{1}$, ${\theta }_{2}$ and ${\theta }_{3}$, which were 0.95, 0.90, and 0.85, respectively. Additionally, the inverse cumulative density function of the uniform distribution was used to generate deterministic equivalent numbers for those chance-constraints (i.e., Eqs. (30) to (32)). For better mirroring of these COVID-19 situations, increased demand from retailer $k$ at period $n$ (i.e., ${\tilde{d }}_{kn}$) were assumed to have three fluctuations (i.e., variations): low variability (e.g., luxury items, outdoor items), medium variability (e.g., clothing, stationery items), and high variability (e.g., home-office items, laptops, and toilet paper). To represent these variabilities, demand from retailer $k$ at period $n$ $(i.e., {d}_{kn})$ was generated by a discretised beta distribution with shape parameters 2 and 5, and an expected value $E({d}_{k})$ equal to the ideal demand value (say, 1000). This demand unit ${\tilde{d }}_{kn}$ has either a low, medium, or high variability, all with equal probability. In the case of low variability, the minimum and maximum values of the stochastic variable ${\tilde{d }}_{kn}$ are equal to $E({d}_{kn})$ and $1.625*E({d}_{kn})$, respectively. In the case of a medium and high variability, the corresponding intervals are $[E\left({d}_{kn}\right), 2.25*E({d}_{kn})]$ and $[E\left({d}_{kn}\right), 2.875*E({d}_{kn})],$ respectively (Deblaere et al., 2011).

### Model complexity

The complexity of any constrained optimization problem can be identified based on the number of decision variables, the number of constraints and their types, and the nature of the objective function. The following set equations were used to calculate the number of decision variables, number of inequality constraints and number of equality constraints for the recovery model.

- Number of variables =|i | ×|j| +|e| ×|j| +|j| +|j| ×|k|
- Number of inequality constraints = N × (|i | +|e| +|j| +|k|)
- Number of equality constraints = N × (|i |+ +|j|)

Here, $|i |$ is the number of suppliers, $\left|j\right|$ the number of manufacturing plants, $|k|$ the number of retailers, $|e|$ the number of emergency suppliers and $N$ is the number of planning periods in the recovery window.

From the abovementioned equations, it is clear any increase in any parameter will lead to an increase in the number of decision variables, number of equality and inequality constraints, which in turn will lead to an increase in the problem complexity. Suppose N = 2, |i |= 3, |e|= 2, |j|= 5 and |k|= 3, then the number of variables is 45; the number of inequality constraints is equal to 26; and the number of equality constraints is equal to 16. If $N$ is changed to 4 and the other parameters remain the same, then the number of inequality constraints becomes 52 and the number of equality constraints becomes 32.

The largest instance we solved was $$15\times 7\times 15\times 8\times 2,$$ which indicated there were 15 different suppliers ($$I$$), 7 different manufacturing plants ($$J)$$, 15 retailers ($$K$$), 8 emergency suppliers ($$e),$$ and 2 planning periods $$(N)$$ in the recovery window.

- Number of variables = 15 × 7 + 8 × 7 + 7 + 7 × 15 = 273
- Number of inequality constraints = 2 × (15 + 7 + 7 + 17) = 98
- Number of equality constraints = 2 × (15 + 7) = 44

From the analysis outlined above, this was already a very complex constrained optimization problem, as it had 142 constraints that the proposed algorithm needed to satisfy to obtain a feasible solution.

### Ideal plan

Table 2 depicts the results of our proposed $ED{E}_{con}$ algorithm for the ideal model. Five different performance measures were reported, as follows: total revenue ($TR$), raw material and transportation cost from suppliers $({RTC}_{s})$, production cost ($PC$), transportation cost from plants to retailers (${TC}_{pr}$), and total profit ($TP$). As shown in Table 2, as $(I,J,K)$ increased, cost components such as ${RTC}_{s}$ and $PC$ also increased. Similarly, the $TP$ also increased with increasing $\left(I,J,K\right),$ which is rational in an SC model under a stable or ideal situation.

**Table 2:** Performance of EDE__con_ for the ideal model

Parameter	3 × 2 × 5	8 × 4 × 10	10 × 6 × 15	15 × 7 × 15
TR	$ 200,000	$ 400,040	$ 599,960	$ 610,800
RTC_S_	$ 25,000	$ 50,005	$ 74,980	$ 76,290
PC	$ 50,000	$ 100,020	$ 149,970	$ 152,890
TC_pr_	$ 50,000	$ 20,002	$ 29,998	$ 30,540
TP	$ 115,000	$ 230,013	$ 345,012	$ 351,080

### Recovery plan

Given the hypothetically considered input data for the recovery model (mentioned in Table 1), the proposed $ED{E}_{con}$ algorithm was utilised to solve the recovery model. Tables 3, 4 and 5 highlight results for all four problem sets. Besides the parameters in the ideal plan (Sect. 5.2), two other cost elements were also added in the recovery model: the cost of increasing capacity $(CIC)$ and the cost of demand lost $(CDL)$. Table 3 highlights the $ED{E}_{con}$ results of a recovery model for varied demand data (i.e., low [L], medium [M], and high [H]), while the belief degrees for Chance-constraints (31) to (33) were considered as 0.95 (i.e., ${\theta }_{1}={\theta }_{2}={\theta }_{3}=0.95$). Similarly, Tables 4 and 5 (in Appendix A) show the results for belief degrees 0.90 and 0.85, respectively.

**Table 3:** Performance of $ED{E}_{con}$ in the recovery model for varied demand data (when ${\theta }_{1}={\theta }_{2}={\theta }_{3}=0.95).$

Parameter	3 × 2 × 5 × 2 × 4			8 × 4 × 10 × 4 × 2		
	L	M	H	L	M	H
TR		$599,840.00	$562,640.00	$716,720	$713,720.00	$743,240.00
RTC_S_	$73,580	$74,980.00	$70,330.00	$89,590	$89,215.00	$92,905.00
RTC_e_	$51,688	$69,440.00	$66,336.00	$46,744	$36,712.00	$57,432.00
PC	$147,160	$149,960.00	140,660	$179,180	$178,430.00	$185,810.00
CIC	$15,290	$17,115.00	$12,245.00	$33,250	$32,830.00	$36,305.00
TC_pr_	$29,432	$29,992.00	$28,132.00	$35,836	$35,686.00	$37,162.00
CDL	$105,700	$100,400.00	$118,840.00	$41,640	$43,040.00	$27,340.00
TP	$165,790	$157,953.00	$126,097.00	$290,480	$297,807.00	$306,286.00

Parameter	10 × 6 × 15 × 6 × 3			15 × 7 × 15 × 8 × 2		
	L	M	H	L	M	H
TR	$1,443,800	$1,649,120.00	$1,560,560	$1,125,440	$1,120,400	$1,124,040
RTC_S_	$180,475	$206,140.00	$195,070	$140,680	$140,050	$140,505
RTC_e_	$165,224	$152,440.00	$167,568	$130,968	$136,792	$119,520
PC	$360,950	$412,280.00	$390,140	$281,360	$280,100	$281,010
CIC	$51,285	$78,525.00	$65,210	$43,290	$41,870	$42,945
TC_pr_	$72,190	$82,456.00	$78,028	$56,272	$56,020	$56,202
CDL	$178,340	$75,440.00	$120,400	$37,580	$38,720	$38,520
TP	$435,336	$641,839.00	$544,144	$435,290	$426,848	$445,338

As can be seen from Table 3, for the 15 × 7 × 15 × 8 × 2 problem set, the $CDL$ values were almost unchanged despite varied demand data due to the higher number of emergency suppliers (i.e., 8).

Similar observations are also presented in Tables 4 and 5 in Appendix A, although their belief degrees were lower than those presented in Table 3. Correspondingly, this can be observed from Table 6, where the $TP$ for any particular demand variation type usually shows lower values for the lower belief degree (i.e., 0.85).

**Table 4:** Impact of belief degrees on total profits (TP) for different problem types

Problem type	TP-L-0.95	TP-M-0.95	TP-H-0.95	TP-L-0.90	TP-M-0.90	TP-H-0.90	TP-L-0.85	TP-M-0.85	TP-H-0.85
3 × 2 × 5 × 2 × 4	165,790	157,953	126,097	92,238	154,367	169,677	144,684	170,644	189,772
8 × 4 × 10 × 4 × 2	290,480	297,807	306,286	236,249	293,404	282,924	305,695	281,064	295,274
10 × 6 × 15 × 6 × 3	435,336	641,839	544,144	493,384	554,939	606,475	523,255	575,489	527,800
15 × 7 × 15 × 8 × 2	435,290	426,848	445,338	427,248	448,607	456,706	480,885	475,574	345,481

**Table 5:** Results of the proposed $ED{E}_{con}$ algorithm for varied planning periods (N) in the recovery window for the $3\times 2\times 5\times 2\times N$ dataset

Planning Periods ($$N$$)	Parameters	$${\theta }_{1}={\theta }_{2}={\theta }_{3}=0.95$$			$${\theta }_{1}={\theta }_{2}={\theta }_{3}=0.90$$			$${\theta }_{1}={\theta }_{2}={\theta }_{3}=0.85$$		
		L	M	H	L	M	H	L	M	H
4	TR	$588,640	$599,840	$562,640	$513,880	$594,760	$618,240	$572,000	$602,280	$613,000
	PC	$147,160	$149,960	$140,660	$128,470	$148,690	$154,560	$143,000	$150,570	$153,250
	CIC	$15,290	$17,115	$12,245	$5,975	$15,960	$19,315	$14,040	$16,995	$18,875
	CDL	$105,700	$100,400	$118,840	$143,300	$103,380	$94,200	$114,400	$98,440	$93,380
	TP	$165,790	$157,953	$126,097	$92,238	$154,367	$169,677	$144,684	$170,644	$189,772
8	TR	$1,130,120	$1,182,120	$1,170,040	$1,242,720	$1,093,360	$1,034,720	$1,144,600	$1,177,520	$1,121,800
	PC	$282,530	$295,530	$292,510	$310,680	$273,340	$258,680	$286,150	$294,380	$280,440
	CIC	$43,850	$56,920	$52,930	$70,250	$35,310	$20,430	$47,690	$58,150	$43,040
	CDL	$235,140	$209,420	$215,420	$190,440	$255,320	$283,600	$228,580	$211,780	$236,920
	TP	$247,229	$310,635	$283,823	$309,335	$236,708	$198,366	$256,803	$281,640	$227,995
10	TR	$1,254,640	$1,331,560	$1,451,320	$1,443,240	$1,332,600	$1,420,000	$1,338,480	$1,348,240	$1,341,200
	PC	$313,650	$332,890	$362,830	$360,810	$333,150	$355,000	$334,630	$337,060	$335,300
	CIC	$15,600	$34,460	$65,150	$62,430	$33,540	$55,600	$35,980	$40,610	$39,630
	CDL	$373,260	$333,060	$277,660	$278,780	$333,340	$291,400	$331,260	$326,120	$329,780
	TP	$161,165	$287,703	$295,691	$373,293	$248,301	$338,252	$228,539	$300,236	$237,266
12	TR	$1,373,240	$1,301,000	$1,398,360	$1,426,400	$1,488,720	$1,358,480	$1,302,400	$1,377,960	$1,407,440
	PC	$343,320	$325,240	$349,590	$356,600	$372,180	$339,620	$325,600	$344,490	$351,860
	CIC	$52,616	$35,600	$64,508	$69,404	$87,824	$48,212	$32,024	$53,552	$62,372
	CDL	$314,320	$350,420	$302,560	$287,380	$254,320	$321,140	$348,520	$312,220	$296,820
	TP	$237,536	$176,491	$325,293	$325,676	$361,294	$278,446	$268,960	$314,995	$286,323

**Fig. 2:**
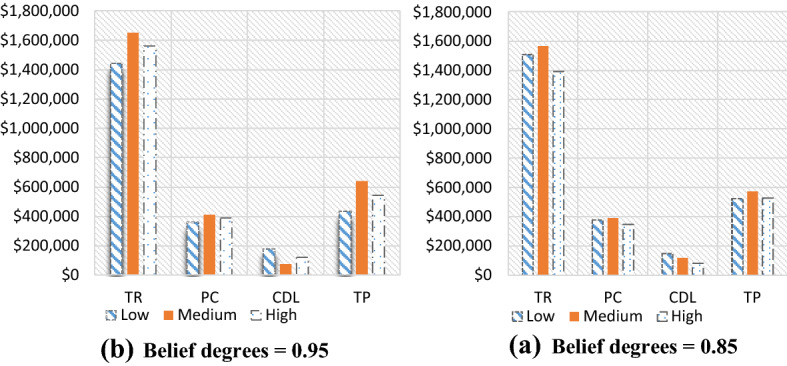
Impact of duration variances on performance parameters of the recovery model for the $10\times 6\times 15\times 6\times 3$ dataset

Figure 2 summarises the impact of duration variabilities on the SC performance parameters, particularly for the 10 × 6 × 15 × 6 × 3 dataset. Specifically, Fig. 2(a) shows the impact of duration variations, wherein the decision-maker imposes 0.95 as the belief degree for all chance-constraints, and Fig. 2(b) uses 0.85.

Notably, the $CDL$ value was higher for the low-demand variability at both the 0.95 and 0.85 belief degrees. This is judicious given that increased demand fluctuations prompt manufacturers to increase the $PC$ and $CIC$ to mitigate the impact of demand variability on the $TR$ and $TP$.

### Sensitivity analysis

In this section, we detail the sensitivity analysis conducted to analyze the impacts of different important parameters.

#### Impact of planning periods (${\varvec{N}}$) in the recovery window

Table 7 summarizes the results of five different performance measures used in our recovery model (i.e., $TR$, $PC, CIC, CDL,$ and $TP$). All input parameters are given in Table 1, while the number of planning periods ($N$) was varied. Four different planning periods were considered (i.e., 4, 8, 10, and 12). Each of these planning periods can be imagined as a month, quarter, semi-annual, or year. For example, if the unit of each of the planning periods is considered a ‘month’, then this table represents the performance of recovery models, while a decision-maker may allocate 12 planning periods during the recovery window (e.g., one year). Table 7 also shows cost and profit data for varied demand scenarios along with varied belief degrees to handle stochastic RHS parameters in the mathematical optimisation model. As can be observed from Table 7, the cost of demand lost ($CDL$) usually increased alongside the value of N (for different belief degree combinations). Similarly, the $CIC$ values also increased with increasing N. This is logical given that higher recovery planning periods mean that manufacturers need to spend more to increase their production capacities. However, the additional production capacity can also increase costs, or the production costs may not have a consequential impact on the $TP$ for larger planning periods. For example, although the values of $CIC$ and $PC$ increased with the increasing N, $TP$ s were not impacted—rather, they showed higher values due to increasing $TR$.

**Fig. 3:**
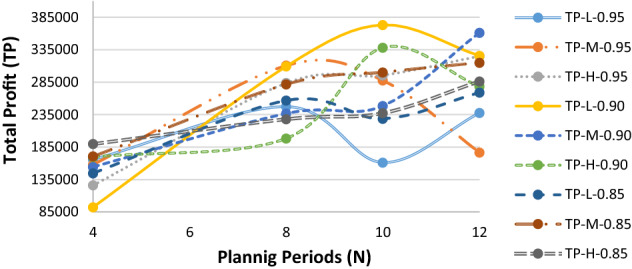
Impact of planning periods (N) on total profits (TP) for the $3\times 2\times 5\times 2\times N$ dataset

Figure 3 briefly illustrates the impact of N on $TP$ for the 3 × 2 × 5 × 2 × N dataset. As evident from this figure, the trend of $TP$ s non-linearly increased up to planning period 8. After this point, it began to decrease, except for the TP-L-0.90, TP-M-0.90, and TP-H-0.90. The $TP$ s, on the other hand, began to decrease for N = 10. Here, TP-L-0.90 represented the $TP$ for the recovery model, while the realised demand data showed lower variability than the expected demand, and the combination of belief degrees was 0.90. Arguably, therefore, for a medium-sized SC recovery model, increasing N values will not always increase profit margins in a linear fashion. Rather, it will decrease if demand variation is high or low, and if the decision-maker’s belief degrees are 0.90 for constraints (31) to (33).

#### Impact of per-unit selling price (S) in the recovery window

**Fig. 4:**
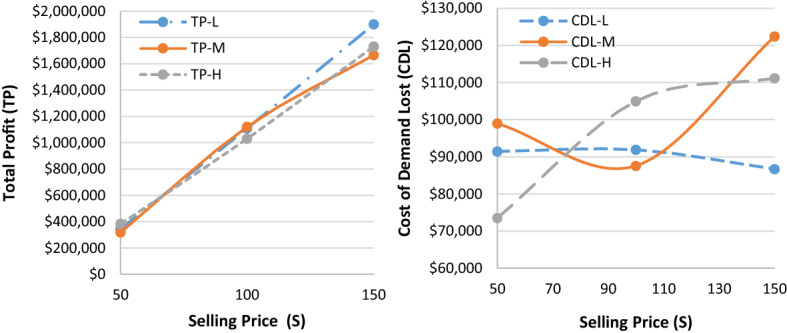
Impact of selling prices on total profit (TP) and cost of demand lost (CDL) for the $3\times 2\times 5\times 2\times 4$ dataset (for belief degree 0.95)

**Fig. 5:**
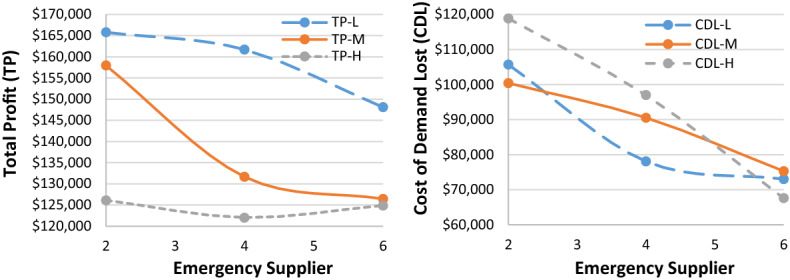
Impact of Selling Prices on Total Profit (TP) and Cost of Demand Lost (CDL) for the 3 × 2 × 5 × 2 × 4 dataset (for belief degrees 0.90 and 0.85)

Figure 4 illustrates the impact of S on both $TP$ and $CDL$ for a medium-sized problem set (i.e., the 3 × 2 × 5 × 2 × 4 dataset). To measure the impact, S values were set as 50, 100, and 150. Figure 4 reports the impact for belief degrees 0.95, while Fig. 5(a) and (b) of Appendix A report the impacts of S when belief degrees were 0.90 and 0.85, respectively. Figure 4 reveals that for the 0.95 belief degrees, the $TP$ increased with increasing S, which was to be expected. While the increment pattern for both TP-H and TP-L was linear, it was non-linear for the TP-M. In contrast, the $CDL$ values showed surprising patterns with the demand fluctuations and S values. CDL values were non-linearly increased with increasing S values, particularly when the demand data showed medium or high variability. Meanwhile, if belief degrees were 0.90, then $CDL$ values decreased after a certain S value (i.e., S = 100), as shown in Fig. 5(a) in Appendix A. Therefore, if the S value is set at more than 100, the manufacturer may experience lower $CDL$. A similar observation was true for that presented in Fig. 5(b) (Appendix A) when belief degrees were equal to 0.85.

#### Impact of per-unit lost sale cost and selling price

Like the impact of S, the per-unit lost sale ($L$) also had an influence on the $TP$ during the recovery period. To observe this impact, we considered three combinations of ($S$, $L$): (50, 25), (50, 50), and (50, 60). The ultimate rationale underpinning such combination selection was to observe the impact of $L$, when it was set at half of the value of $S$, equal to S and a bit higher than $S$. Table 8 summarises all results obtained from the proposed ${EDE}_{con}$ approach with varied *S, L*, demand, and belief degrees. As expected, increasing the *L* values from 25 to 60 (without changing the *S* values) decreased the $TP$ margin for all demand fluctuation scenarios and all belief degree combinations. Therefore, the recovery model always emphasises minimising the gap between actual demands and demand fulfilment (i.e., total delivery to all retailers). Consequently, the $CDL$ values increased with increasing *L* values, which was also an expected outcome.

**Table 6:** Performance of the proposed $ED{E}_{con}$ for varied selling price and lost sales for the $3\times 2\times 5\times 2\times 4$ dataset

(S, L)	Parameters	$${\theta }_{1}={\theta }_{2}={\theta }_{3}=0.95$$			$${\theta }_{1}={\theta }_{2}={\theta }_{3}=0.90$$			$${\theta }_{1}={\theta }_{2}={\theta }_{3}=0.85$$		
		L	M	H	L	M	H	L	M	H
(50,25)	TR	$719,850	$734,000	$737,500	$773,300	$735,150	$792,950	$752,850	$753,850	$744,550
	PC	$143,970	$146,800	$147,500	$154,660	$147,030	$158,590	$150,570	$150,770	$148,910
	CIC	$14,135	$15,180	$15,750	$19,525	$15,760	$20,535	$16,695	$17,440	$16,425
	CDL	$140,850	$133,775	$130,025	$113,250	$133,000	$105,300	$125,050	$122,750	$128,300
	TP	$257,692	$271,965	$286,471	$322,499	$277,551	$342,376	$294,304	$299,647	$295,382
(50,50)	TR	$780,000	$789,300	$831,500	$787,400	$760,650	$770,350	$769,100	$774,300	$770,600
	PC	$156,000	$157,860	$166,300	$157,480	$152,130	$154,070	$153,820	$154,860	$154,120
	CIC	$19,685	$21,060	$25,145	$20,810	$18,535	$19,245	$19,315	$18,770	$19,050
	CDL	$222,150	$206,450	$170,750	$214,350	$240,050	$229,500	$232,050	$225,500	$232,550
	TP	$218,693	$247,132	$301,135	$239,924	$191,564	$206,462	$201,297	$218,672	$203,244
(50,60)	TR	$770,550	$794,100	$813,450	$819,200	$774,750	$804,050	$765,550	$763,950	$777,000
	PC	$154,110	$158,820	$162,690	$163,840	$154,950	$160,810	$153,110	$152,790	$155,400
	CIC	$18,850	$21,490	$23,050	$23,860	$19,345	$22,535	$18,660	$18,800	$19,550
	CDL	$275,160	$245,700	$230,400	$217,860	$273,480	$234,480	$282,600	$285,840	$270,840
	TP	$164,577	$201,260	$223,747	$255,816	$169,190	$220,442	$148,955	$145,583	$178,358

#### Impact of the number of emergency suppliers

**Fig. 6:**
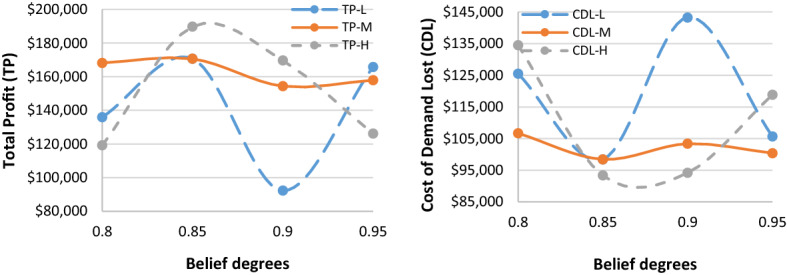
Impact of the number of emergency suppliers on total profit (TP) and cost of demand lost (CDL) for the $3\times 2\times 5\times 2\times 4$ dataset (for belief degree 0.95)

**Fig. 7:**
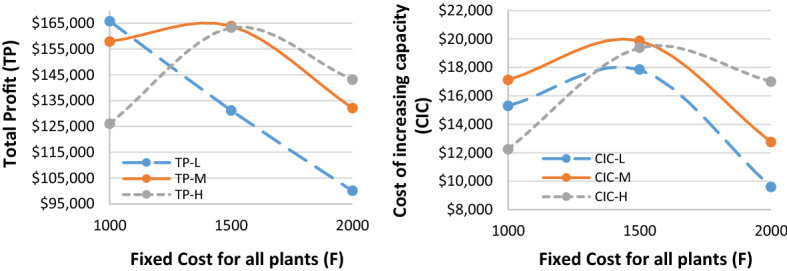
Impact of the number of emergency suppliers on Total Profit (TP) and Cost of Demand Lost (CDL) for the 3 × 2 × 5 × 2 × 4 dataset (for belief degrees 0.90 and 0.85)

During the pandemic period, due to varied (but increased) demand fluctuations and compulsory social distancing measures, suppliers’ capacities have been vulnerable to change or reduction. In this challenging situation, manufacturers or plants are highly dependent on the number of emergency suppliers and their capacities. As expected, it was found that if a plant has a good number of emergency suppliers as a backup, its sustainability is also high due to increased opportunities to subcontract unmet demands. To observe the impact of ‘the number of emergency supplier’s (e), this study considered three different values of e: 2, 4, and 6. Figures 6, 7(a), and (b) report the results of ${EDE}_{con}$ for varied e values for the recovery model of the $3\times 2\times 5\times 2\times 4$ problem set, while the belief degrees were 0.95, 0.90, and 0.85, respectively. Next, as evident from Figs. 6 and 7(a), when the belief degrees were comparatively higher, the $CDL$ values decreased with increasing e. This is logical, as with increasing emergency supplier numbers, plants can subcontract to minimise unmet demands. However, when practitioners have less confidence over their capacity constraints (i.e., belief degrees equal 0.85), $CDL$ values were found to increase up to a certain number of e (here, at 4) and then began decreasing, particularly when demand data fluctuations were medium or high (shown in Fig. 7(b)). Although the $CDL$ decreased with increasing $e$ values, the $TP$ values were also decreasing exceptionally, for almost all cases. Essentially, if $CDL$ values are low, $TP$ should be higher. Nevertheless, the $TP$ values decreased, mostly because of increases in other SC costs (e.g., ${RTC}_{e}, PC, CIC,$ and ${TC}_{pr}$). Notably, Fig. 7 is given in Appendix A.

#### Impact of belief degrees

**Fig. 8:**
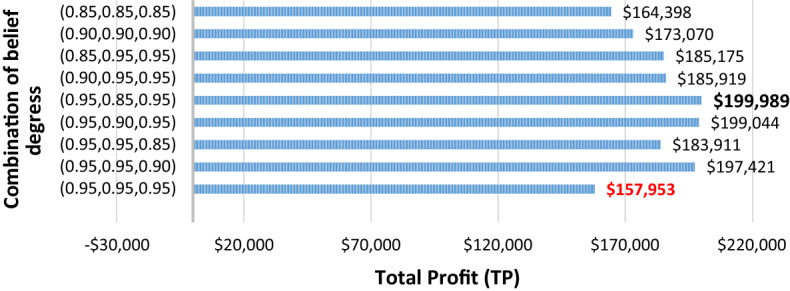
Impact of belief degrees on total profit (TP) and cost of demand lost (CDL) for the $3\times 2\times 5\times 2\times 4$ dataset

Indisputably, the confidence interval or belief degrees should have a significant impact on the mathematical optimisation approach, and thus on the model’s performance parameters. To measure this impact, we considered four different belief degrees (0.95, 0.90, 0.85, and 0.80). The results obtained after applying the proposed ${EDE}_{con}$ approach for the 3 × 2 × 5 × 2 × 4 dataset are given in Table 9 (Appendix A). As discussed in chance-constraint Eqs. (32) to (34), if a ${\theta }_{1}$ value is 0.95, then the model needs to satisfy that for at least 95% cases, where the chance constraint is then satisfied. Meanwhile, for lower ${\theta }_{1}$ values, the chance constraint is less strict. As observed from Fig. 8, the $TP$ values increased from 0.80 to 0.85, then began to drop from 0.85 to 0.90, and then again increased from 0.90 to 0.95 (except for the TP-H). Therefore, the impact of belief degrees on multiple chance-constraints was difficult to predict and did not show any linear trend. It can further be claimed that if the belief degrees are 0.85 and the demand data shows high variability, then the $TP$ will be the highest. Additionally, the impact of belief degrees on the $CDL$ was also non-linear.

We further conducted a sensitivity analysis for different parameters and observed that the model reacted very well.

#### Impact of fixed costs of increasing production capacity

**Fig. 9:**
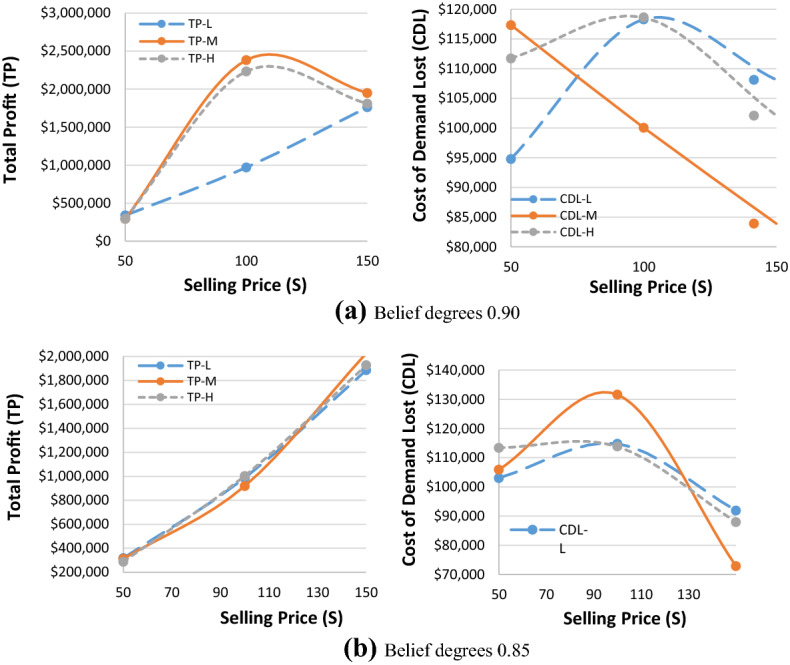
Impact of fixed costs of increasing production capacity (F) in the recovery period for the $3\times 2\times 5\times 2\times 4$ dataset (${{\varvec{\theta}}}_{1}={{\varvec{\theta}}}_{2}={{\varvec{\theta}}}_{3}=0.95$

Due to the increasing demand during pandemics, plants or manufacturers are forced to increase their production capacity to minimise the gap of unmet demands. However, increasing production capacity largely depends on the number of normal and emergency suppliers and their stochastic capacity and the availability of transportation materials. Now, to observe the impact of the fixed cost of increasing production capacity (F), this study considered three different values of F for the 3 × 2 × 5 × 2 × 4 problem set: $1000, $1500, and $2000. Three demand fluctuations and belief degrees of 0.95 were considered to solve this problem set. Notably, both plants were assumed to have the same F values. As expected, Fig. 9 shows that the $$TPs$$ decreased with increasing $$F$$ values. However, the rate of decrement is non-linear, solely due to the influence of other parameters (e.g., operating costs, variable costs, transportation costs, and project-specific costs). Furthermore, as illustrated in Fig. 9, the $$CIC$$ increased with increasing $$F$$ values up to 1500. After this point, the cost began to decrease. Thus, the impact of a fixed cost is nullified once the production capacity has reached a certain level.

#### Impact of different belief degree combinations

**Fig. 10:**
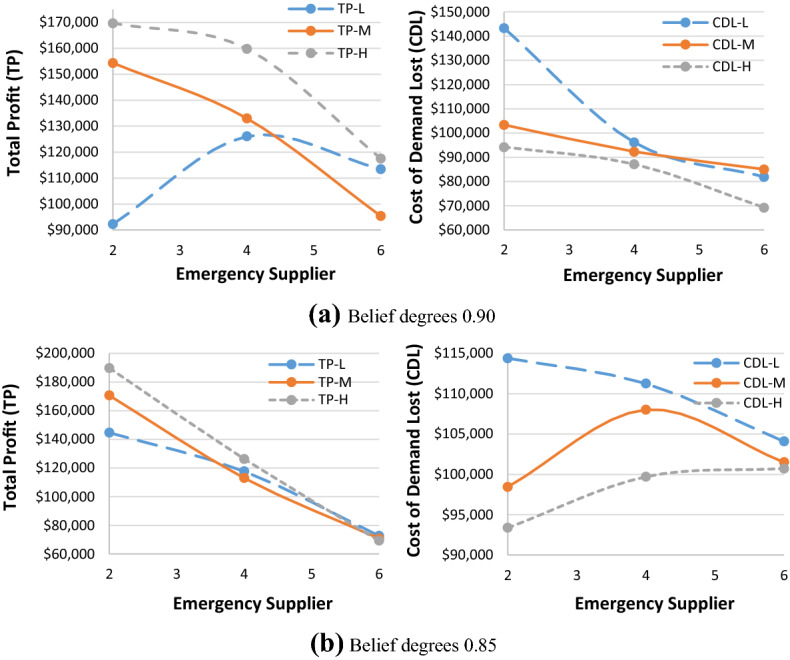
Impact of different belief degree combinations on the total profit in the recovery period for the 3 × 2 × 5 × 2 × 4 dataset (for HIGH duration variance only)

Throughout all of the numerical experiments, the belief degrees for all three Chance-constraint Eqs. (32) to (34) were considered equal (i.e., ${\theta }_{1}={\theta }_{2}={\theta }_{3}).$ However, in practice, the belief degree may vary for different uncertain RHS parameters. For example, the manufacturer may have higher confidence in the variability of production capacity and supplier capacity but lower confidence in the emergency supplier’s capacity. In that case, ${\theta }_{1}$ and ${\theta }_{3}$ should be higher than ${\theta }_{2}$. Therefore, a set of experiments was needed to demonstrate the impact of varied belief degrees. Consequently, this work considered nine different combinations of ${\theta }_{1}, {\theta }_{2}$ and ${\theta }_{3}$, as shown in Fig. 10.

The results were obtained after employing the proposed ${EDE}_{con}$ meta-heuristic algorithm for the 3 × 2 × 5 × 2 × 4 problem set. For simplicity, only the high duration variance was considered. As evident from Fig. 5, the maximum value of $TP$ was obtained when the combination was (0.95, 0.85, 0.95). Therefore, if the decision-maker (i.e., manufacturer) has high belief degrees for dealing with stochastic production capacity and stochastic supplier capacity and a low belief degree for the stochastic emergency supplier capacity, then the resultant $TP$ value will be higher. However, if all of these belief degrees are at the highest level (0.95), then the recovery model will produce the least $TP$.

### Managerial insights

From Table 3 it can be seen that the $$TP$$ (bolded values) for problem set $$3\times 2\times 5\times 2\times 4$$ showed varied values along with the varied demand data. For instance, if the demand showed low variability in the expected or ideal model’s demand data, then the $$TP$$ equalled $165,790. If the demand showed medium variability, then the $$TP$$ dropped to $157,953. For high-demand variability, the $$TP$$ dropped even further to $126,097. In contrast, for the larger SC design, particularly if the number of emergency suppliers was comparatively higher (e.g., for the 15 × 7 × 15 × 8 × 2 problem set), the impact of demand variabilities on the $$TP$$ were not practically significant. Further, they did not show any particular trend of increasing or decreasing with the demand variability. The following managerial insight 1 can be drawn from this finding.

***Managerial insight 1:*** The impact of demand variability significantly contributes to shrinking profit margins, particularly for smaller SC designs (i.e., when the number of suppliers, plants, and retailers is low). In larger SC networks, manufacturers have more flexibility to order shortage materials from their emergency suppliers and to minimise the cost of demand lost.

Table 6 provides a detailed discussion on the impact of different belief degrees on $TP$. Essentially, a higher value of θ means that the service level is increased, and hence the decision-maker will want to satisfy the original ‘stochastic’ constraint with a higher probability. Hence, the chance constraint needs to become stricter when θ increases. So, with lower belief degrees, the chance-constraints are pragmatically easier to solve, which provides the model with less control over limiting variables (e.g., decision variables), and thus often produces lower $TP$ (or higher costs). This is obvious since the decision-maker has less confidence in the controlling parameters of RHS uncertain values (Zhang et al., 2016). Also, Fig. 2 shows the impact of duration variabilities on the SC performance parameters. From these findings, the following managerial insight 2 can be drawn.

***Managerial insight 2:*** TR and TP are higher for the medium variability demand data. With increasing variability from medium to high, the TP and TR values are decreased, solely due to increased uncertainty in the SC model. However, the change in profit margins does not follow the same pattern if the variability of demand data is increased from low to medium.

In this study, we used hypothetical and random data. However, in practice, these data can be estimated using historical demand, supply, and cost information. Also, decision-maker can use emergent technologies such as blockchain, artificial intelligence, and Industry 4.0 to store data and information which can be used in the model to develop the recovery plan (Ivanov et al., 2022; Dolgui and Ivanov 2022; Ivanov 2021d).

## Conclusions

The impacts of COVID-19 on SCs are devastating and have spread across different network stages. The high-demand and essential product SCs have predominantly been significantly affected by the pandemic due to its multi-dimensional impacts, such as demand surge and reduction in supply and production capacities, wherein the extent of variations is uncertain. Without proper planning and strategies, companies may lose substantial demand and, therefore, profit in the short-term, potentially facing complete shutdown in the medium- to long-term.

We took up this issue and studied the recovery of an SC for a high-demand item (e.g., hand sanitizers or face masks). We began by defining a stochastic mathematical model to optimise a recovery plan in a three-stage SC facing the multi-dimensional impacts of the COVID-19 pandemic. In this setting, we developed a constrained programming mathematical model that optimises the total SC profit in the recovery window by considering the multi-dimensional yet uncertain impacts of a pandemic. Our definition generalizes a unique problem setting with simultaneous demand, supply, and capacity uncertainties in a multi-stage SC recovery context. In the mathematical model, we considered multiple strategies that can simultaneously enhance the production capacity and raw material supply. The model also considered the cost of lost sales if firms are unable to meet the demand or perceive that sacrificing sales would be more profitable than increasing capacity to meet the short-term and sudden demand. This feature of the model enables practitioners to decide between increasing capacities to meet demand and sacrificing sales. The findings revealed that companies could significantly improve their total profit by implementing the strategies suggested for various scenarios and the model developed in this study.

We then presented a new, enhanced multi-operator differential evolution variant-based solution approach to solve our model. Through extensive numerical experiments, we demonstrated how the solution approach developed is capable of solving both small- and large-scale SC recovery problems. With the optimisation, we sought to understand the impact of different recovery strategies on SC profitability and to determine the optimal recovery plans through adjustments of supply, production, and transportation quantities. We tested, evaluated, and analysed different recovery strategies, scenarios, and problem scales. Ultimately, we provide a useful tool to optimize reactive adaptation strategies related to how and when to recover the SC operations during a pandemic. The outcomes of this research can be used by decision-makers to select the most efficient SC recovery plan in a pandemic setting and to determine the timing of its deployment.

Despite its substantial contributions, the study also has some limitations that must be acknowledged. For example, the SC recovery model developed in this study is effective for planning recovery strategies for a high-demand item. However, the COVID-19 pandemic has simultaneously affected the SCs of multiple high-demand items as well as several high-demand and stable demand products. In such a situation, a modified SC recovery model, which is capable of considering recovery plans for multiple products and intertwined supply networks, is needed. Therefore, future studies could work to develop such models. This study also focused exclusively on a manufacturing plant, its immediate tier-one suppliers, and downstream retailers. In the real world, however, an SC is typically more complex, and many other members (such as upstream suppliers at various tiers) and middlemen (such as distributors, brokers, and wholesalers) are also involved in the operations. Therefore, future studies could develop models that consider all potential SC members to enhance the applicability of the model. Finally, this study considered hypothetical, yet realistic input data for analyzing the various scenarios within the model. Future studies could provide a further empirical examination via multiple case studies or a large-scale survey to explore and test the benefits of the strategies suggested in this research to enhance their generalizability. Considering the relevance of including sustainability features (e.g., social impact and environmental sustainability) with economical and resilience objectives, another possible extension of this work could be to consider multi-objective formulations.

## Appendix A

See (Figs. 9 and 10) .

(Tables 7, 8 and 9).

**Table 7:** Performance of $ED{E}_{con}$ in the recovery model for varied demand data (when ${\theta }_{1}={\theta }_{2}={\theta }_{3}=0.90)$

Parameter	3 × 2 × 5 × 2 × 4			8 × 4 × 10 × 4 × 2		
	L	M	H	L	M	H
TR	$513,880	$594,760	$618,240	$662,280	$720,200	$741,240
RTCS	$64,235	$74,345	$77,280	$82,785	$90,025	$92,655
RTCE	$53,968	$68,280	$72,296	$49,032	$48,536	$77,264
PC	$128,470	$148,690	$154,560	$165,570	$180,050	$185,310
CIC	$5,975	$15,960	$19,315	$27,010	$34,235	$36,545
TC_pr_	$25,694	$29,738	$30,912	$33,114	$36,010	$37,062
CDL	$143,300	$103,380	$94,200	$68,520	$37,940	$29,480
TP	$92,238	$154,367	$169,677	$236,249	$293,404	$282,924

Parameter	10 × 6 × 15 × 6 × 3			15 × 7 × 15 × 8 × 2		
	L	M	H	L	M	H
TR	$1,470,600	$1,527,560	$1,606,440	$1,110,360	$1,139,120	$1,156,320
RTCS	$183,825	$190,945	$200,805	$138,795	$142,390	$144,540
RTCE	$132,176	$125,968	$145,648	$125,024	$131,232	$138,448
PC	$367,650	$381,890	$401,610	$277,590	$284,780	$289,080
CIC	$54,915	$61,500	$72,160	$40,745	$44,355	$46,930
TC_pr_	$73,530	$76,378	$80,322	$55,518	$56,956	$57,816
CDL	$165,120	$135,940	$99,420	$45,440	$30,800	$22,800
TP	$493,384	$554,939	$606,475	$427,248	$448,607	$456,706

**Table 8:** Performance of $ED{E}_{con}$ in the recovery model for varied demand data (when ${\theta }_{1}={\theta }_{2}={\theta }_{3}=0.85)$

Parameter	3 × 2 × 5 × 2 × 4			8 × 4 × 10 × 4 × 2		
	L	M	H	L	M	H
TR	$572,000	$602,280	$613,000	$734,040	$711,200	$733,080
RTC_S_	$71,500	$75,285	$76,625	$91,755	$88,900	$91,635
RTC_E_	$56,776	$60,232	$50,448	$48,688	$51,456	$55,432
PC	$143,000	$150,570	$153,250	$183,510	$177,800	$183,270
CIC	$14,040	$16,995	$18,875	$35,050	$32,400	$36,055
TC_pr_	$28,600	$30,114	$30,650	$36,702	$35,560	$36,654
CDL	$114,400	$98,440	$93,380	$32,640	$44,020	$34,760
TP	$144,684	$170,644	$189,772	$305,695	$281,064	$295,274

Parameter	$10\times 6\times 15\times 6\times 3$			$15\times 7\times 15\times 8\times 2$		
	L	M	H	L	M	H
TR	$1,508,680	$1,565,640	$1,391,720	$1,144,680	$1,144,360	$1,013,920
RTC_S_	$188,585	$195,705	$173,965	$143,085	$143,045	$126,740
RTC_E_	$138,816	$139,104	$146,392	$103,936	$110,928	$117,848
PC	$377,170	$391,410	$347,930	$286,170	$286,090	$253,480
CIC	$58,580	$67,130	$44,925	$45,750	$44,705	$28,755
TC_pr_	$75,434	$78,282	$69,586	$57,234	$57,218	$50,696
CDL	$146,840	$118,520	$81,122	$27,620	$26,800	$90,920
TP	$523,255	$575,489	$527,800	$480,885	$475,574	$345,481

**Table 9:** Performance for varied belief degrees for the $3\times 2\times 5\times 2\times 4$ dataset

Parameters	${\theta }_{1}={\theta }_{2}={\theta }_{3}=0.95$			${\theta }_{1}={\theta }_{2}={\theta }_{3}=0.90$		
	L	M	H	L	M	H
TR	$588,640	$599,840	$562,640	$513,880	$594,760	$618,240
PC	$147,160	$149,960	$140,660	$128,470	$148,690	$154,560
CIC	$15,290	$17,115	$12,245	$5,975	$15,960	$19,315
CDL	$105,700	$100,400	$118,840	$143,300	$103,380	$94,200
TP	$165,790	$157,953	$126,097	$92,238	$154,367	$169,677

Parameters	${\theta }_{1}={\theta }_{2}={\theta }_{3}=0.85$			${\theta }_{1}={\theta }_{2}={\theta }_{3}=0.80$		
	L	M	H	L	M	H
TR	$572,000	$602,280	$613,000	$548,720	$586,360	$535,320
PC	$143,000	$150,570	$153,250	$137,180	$146,590	$133,830
CIC	$14,040	$16,995	$18,875	$10,810	$15,465	$8,490
CDL	$114,400	$98,440	$93,380	$125,540	$106,700	$134,540
TP	$144,684	$170,644	$189,772	$135,948	$168,224	$119,115
